# Supplementary material for: Resin acids play key roles in shaping microbial communities during degradation of spruce bark
Source: Nat Commun. 2023 Dec 9;14:8171. doi: 10.1038/s41467-023-43867-y (PMC10710418; doi:10.1038/s41467-023-43867-y)
Supplement: Supplementary file 1 — Supplementary Information [file 41467_2023_43867_MOESM1_ESM.pdf]

Supplemental information

Resin acids play key roles in shaping microbial communities during degradation of spruce bark

Authors: Amanda Sörensen Ristinmaa[1], Albert Tafur Rangel[1,2], Alexander Idström[3], Sebastian Valenzuela[4], Eduard J Kerkhoven[1,2], Phillip B. Pope[5,6], Merima Hasani[2,7], and Johan Larsbrink[1,7*]

Affiliations:

[1]Department of Life Sciences, Chalmers University of Technology, SE-412 96 Gothenburg, Sweden

[2]Novo Nordisk Foundation Center for Biosustainability, Technical University of Denmark, DK-2800 Kgs. Lyngby, Denmark

[3]Department Chemistry and Chemical Engineering, Chalmers University of Technology, SE-412 96 Gothenburg, Sweden

[4]Department of Medical Biochemistry and Cell Biology, University of Gothenburg, SE-405 30 Gothenburg, Sweden

[5]Faculty of Biosciences, Norwegian University of Life Sciences, NO-1433 Ås, Norway

[6]Faculty of Chemistry, Biotechnology and Food Science, Norwegian University of Life Sciences, NO-1433 Ås, Norway

[7]Wallenberg Wood Science Center, Chalmers University of Technology, SE-412 96 Gothenburg, Sweden

*To whom correspondence should be addressed, at johan.larsbrink@chalmers.se

INDEX

Supplementary tables 3

Supplementary figures 14

Supplementary References 38

Supplementary tables

Table S1. Intervals of integration for the main classes of extractive compounds and internal standards in the spruce bark extractives using GC-MS and the corresponding column used.

**Class of compounds**			
Name	Acronym	Retention time interval (min)	Column (length)
Fatty acids	FA	14.5-16.9	HP-5ms (30 m)
Resin acids	RA	17.0-18.0	HP-5ms (30 m)
Sterols	ST	24.1-25.6	HP-5ms (30 m)
Steryl esters	SE	17.3-18.9	HP-1 SIM/Dist(5 m)
Triglycerides	TG	19.7-22.0	HP-1 SIM/Dist (5 m)
**Internal standards**			
Name	Acronym	Retention time interval (min)	Column (length)
Heptadecaonic acid	INS1	20.05 – 20.14	HP-5ms (30 m)
Cholesterylheptadecanoate	INS2	16.9-17.4	HP-1 SIM/Dist(5 m)
1.3-dipalmitoyl-2 oleyl glycerol	INS3	18.9-19.7	HP-1 SIM/Dist(5 m)

Table S2. Identification of individual compounds in spruce bark extract by GC-MS/FID on a HP-5ms column after derivatization with BSTFA/TMCS/pyridine in the biotic sample at two weeks growth, and the abiotic sample at week zero.

Retention time (min)	Compound name	CAS nr	Formula	Match factor	Reverse match	Probability (%)
14.2	Hexadecanoic acid	57-10-3	C16H32O2	789	826	65
14.85	Heptadecanoic acid	506-12-7	C17H34O2	705	854	57.5
15.54	9,12,15-Octadecatrienoic acid	1955-33-5	C18H30O2 (C18:3)	803	843	20.8
15.702	9,12-Octadecadienoic acid	2197-37-7	C18H34O2 (C18:2)	882	947	63.4
15.745	(9,Z)-Octadec-9-enoic acid	112-80-1	C18H34O2 (C18:1)	897	943	22.8
15.955	Stearic acid		C18	497	705	24.7
16.655	Pimaric acid	127-27-5	C20H30O2	861	887	72.8
16.785	Isopimaric acid	5835-26-7	C20H30O2	868	911	66.6
17.075	Dehydroabietic acid	1740-19-8	C20H28O2	816	835	71.9
17.54	Abietic acid	514-10-3	C20H30O2	868	889	82.3
19.098	7-oxodehydroabietic acid	18684-55-4	C20H26O3	783	836	91.7

*900 or greater is considered an excellent match; 800–900 a good match; 700–800 a fair match and less than 600 is a very poor match

Table S3. Sequencing statistics for both bacteria and fungi. Extraction conc. is the concentration of the extracted DNA (ng/μL). Library conc. is the concentration of the sequencing library (ng/μL). Reads is the number of DNA reads after sequencing, QC and bioinformatic processing. Observed OTUs is the number of observed, unique OTUs in each sample.

libType	**Sample name**	**Extraction concentration (ng/uL)**	**Library conc (ng/uL)**	**Reads**	**Observed OTUs**
bV18-A	w0-s1	7.76	8.3	49024	642
bV18-A	w0-s3	6.06	7.7	60959	695
bV18-A	w2-s5	114	21.3	56976	425
bV18-A	w2-s6	110	34.6	67759	422
bV18-A	w4-s8	120	19.1	76668	521
bV18-A	w4-s9	104	19.5	74223	566
bV18-A	w8-s11	68.8	24.4	74634	552
bV18-A	w8-s12	102	22.8	57570	501
bV18-A	w12-s14	93.2	12.7	82576	591
bV18-A	w12-s15	97.8	12.4	52945	630
bV18-A	w16-s17	102	5.2	31959	431
bV18-A	w16-s18	25.4	11.5	21047	541
bV18-A	w20-s20	69.4	12.9	80473	619
bV18-A	w20-s21	70.2	18.1	79863	718
bV18-A	w24-s23	16	13.5	77010	584
bV18-A	w24-s24	55.6	11.7	82926	577
fITS2-C	w0-s1	7.76	2.5	86969	200
fITS2-C	w0-s3	6.06	1.9	72491	197
fITS2-C	w2-s5	114	13.6	97559	141
fITS2-C	w2-s6	110	23.2	84546	136
fITS2-C	w4-s8	120	34.4	99041	131
fITS2-C	w4-s9	104	31.8	37049	144
fITS2-C	w8-s11	68.8	27.9	44844	136
fITS2-C	w8-s12	102	30.7	99085	137
fITS2-C	w12-s14	93.2	25	99130	144
fITS2-C	w12-s15	97.8	21.2	98979	137
fITS2-C	w16-s17	102	26.5	99000	140
fITS2-C	w16-s18	25.4	25	98848	132
fITS2-C	w20-s20	69.4	24.6	98638	128
fITS2-C	w20-s21	70.2	24.3	98741	147
fITS2-C	w24-s23	16	19.3	99232	131
fITS2-C	w24-s24	55.6	19.7	82495	122

Table S4. Bark two-week metagenome assembly statistics. The number of contigs denotes the number of de novo assembled contigs, with length total (Mb) denoting the total length (in megabases). Length maximum (Mb), length mean (bp) and length minimum (bp) denote the maximum, mean and minimum contig lengths (in basepairs). N50 (bp) is defined as the sequence length of the shortest contig at 50% of the total assembly length. Mean GC content (%) denotes the mean content of the G- and C-nucleotides in the assembly.

Summary	Bark assembly
Number of contigs	15,474
Total length (Mb)	430
Length maximum (Mb)	4.32
Length mean (bp)	27,784
Length minimum (bp)	344
N50 (bp)	49,770
Mean GC content (%)	59
Data (Gbp) - Oxford Nanopore	16
Reads (mio.) - Oxford Nanopore	2.9
Read N50 - Oxford Nanopore	6,558
Q-score - Oxford Nanopore	14.5
Data (Gbp) - Illumina	340
Reads (mio.) - Illumina	2,266
Q-score - Illumina	33.8

Table S5. MAG statistics. MAG ID and MAG size refer to the bin identification number and the size of the MAG in megabases, respectively. Contigs denotes the number of contiguous DNA elements associated with each MAG. Compl. (%) is the estimated genome completeness based on the presence/absence of essential lineage-specific marker genes. Cont. (%) is the estimated contamination based on the presence of multiple single-copy marker genes. N50 (mb) is defined as the sequence length (megabases) of the shortest contig at 50% of the total MAG length. MAG quality refers to the quality of the extracted genome based on the MIMAG standard (Minimal Information about a Metagenome-assembled genome), with HQ denoting high-quality (>90% completion, <5% contamination, presence of 5S, 16S and 23S rRNA and a minimum of 18 tRNA detected) MAGs. GTDB taxonomy refers to the GTDB (Genome Taxonomy Database) classification at the highest taxonomic resolution assigned to a specific MAG.

MAG ID	MAG size (mbp)	contigs	N50 (mbp)	Compl (%)	Cont (%)	GTDB taxonomy	MAG quality
Bin1	7.16	5	2.21	99.34	0.15	*Nitrospirillum*	HQ
Bin2	4.32	1	4.32	98.45	2.43	*Pseudoxanthomonas*_A	HQ
Bin3	2.56	9	1.42	98.44	1.69	GAS474	HQ
Bin4	3.24	9	2.08	99.35	0.10	JAATFR01	HQ
Bin5	3.37	10	0.38	95.66	8.41	UBA2020	MQ
Bin6	8.09	8	1.40	96.51	1.56	*Paraburkholderia tropica*	HQ
Bin7	7.06	21	0.45	97.47	1.69	*Paraburkholderia*	HQ
Bin8	4.09	4	1.10	99.61	0.00	*Neorhizobium*	HQ
Bin9	6.24	34	0.34	98.39	1.23	*Pantoea*	HQ
Bin10	4.27	5	3.59	100.00	0.86	Tous-C9LFEB	HQ
Bin11	4.79	10	0.56	96.55	0.86	*Edaphobacter*	HQ
Bin12	3.01	31	0.19	90.52	2.76	Tous-C9LFEB	MQ
Bin13	3.59	35	0.13	97.78	1.72	*Arachidicoccus*	HQ
Bin14	6.45	16	0.61	99.18	1.19	*Pseudomonas*_E sp002080045	HQ
Bin15	6.62	69	0.16	90.67	3.08	*Pseudomonas*_E	HQ

Table S6. NCBI accession number of the genes from the *dit* cluster genes from *Pseudomonas abietaniphila* BKME-9^T and *Paraburkholderia xenovorans* LB400^T.

Gene	NBCI accession number	Description	Microorganism
ditI	AAD21071.1	Dehydrogenase/reductase	*Pseudomonas abietaniphila* BKME-9[1]
ditA2	AAD21061.1	β Subunit of the ring-hydroxylating dioxygenase	*Pseudomonas abietaniphila* BKME-9[1]
ditA1	AAD21063.1	α Subunit of the ring-hydroxylating dioxygenase	*Pseudomonas abietaniphila* BKME-9[1]
ditH	AAD21070.1	Isomerase/decarboxylase	*Pseudomonas abietaniphila* BKME-9[1]
ditG	AAD21069.1	Dehydrogenase/reductase	*Pseudomonas abietaniphila* BKME-9[1]
ditF	AAD21068.1	Sterol carrier-like protein	*Pseudomonas abietaniphila* BKME-9[1]
ditR	AAD21072.1	IclR-type transcription regulator	*Pseudomonas abietaniphila* BKME-9[1]
ditE	AAD21067.1	Permease of the major facilitator superfamily	*Pseudomonas abietaniphila* BKME-9[1]
ditD	AAD21066.1	Isomerase/decarboxylase	*Pseudomonas abietaniphila* BKME-9[1]
ditC	AAD21065.1	Extradiol cleavage dioxygenase	*Pseudomonas abietaniphila* BKME-9[1]
ditB	AAD21064.1	Dehydrogenase/reductase	*Pseudomonas abietaniphila* BKME-9[1]
ORF2	AAD21074.1		*Pseudomonas abietaniphila* BKME-9[1]
ditA3	AAD21062.1	Ferredoxin component of ring-hydroxylating dioxygenase	*Pseudomonas abietaniphila* BKME-9[1]
ORF3	AAR83736.1		*Pseudomonas abietaniphila* BKME-9[2]
ORF4	AAR83737.1		*Pseudomonas abietaniphila* BKME-9[2]
ditJ	AAD21073.2	CoA ligase	*Pseudomonas abietaniphila* BKME-9[2]
ditK	AAR83744.1	Transcriptional regulator, TetR family	*Pseudomonas abietaniphila* BKME-9[2]
ditL	AAR83743.1	Hypothetical protein	*Pseudomonas abietaniphila* BKME-9[2]
ditM	AAR83742.1	Hydrolase	*Pseudomonas abietaniphila* BKME-9[2]
ditN	AAR83741.1	3-hydroxyacyl CoA dehydrogenase	*Pseudomonas abietaniphila* BKME-9
ditO	AAR83740.1	Thiolase	*Pseudomonas abietaniphila* BKME-9[2]
ditP	AAR83739.1	Conserved hypothetical protein	*Pseudomonas abietaniphila* BKME-9[2]
ditQ	AAR83738.1	Cytochrome P450	*Pseudomonas abietaniphila* BKME-9[2]
ditA2	ABE36485.1	β Subunit of the ring-hydroxylating dioxygenase	*Paraburkholderia xenovorans* LB400[3]
ditA1	ABE36484.1	α Subunit of the ring-hydroxylating dioxygenase	*Paraburkholderia xenovorans* LB400[3]
ditH	ABE36483.1	Isomerase/decarboxylase	*Paraburkholderia xenovorans* LB400[3]
ditG	ABE36481.1	Dehydrogenase/reductase	*Paraburkholderia xenovorans* LB400[3]

ditF	ABE36479.1	Sterol carrier-like protein	*Paraburkholderia xenovorans* LB400[3]
ditR	ABE36505.1	IclR-type transcription regulator	*Paraburkholderia xenovorans* LB400[3]
ditD	ABE36506.1	Isomerase/decarboxylase	*Paraburkholderia xenovorans* LB400[3]
ditC	ABE36538.1	Extradiol cleavage dioxygenase	*Paraburkholderia xenovorans* LB400[3]
ditB	ABE36537.1	Dehydrogenase/reductase	*Paraburkholderia xenovorans* LB400[3]
ditA3	ABE36536.1	Ferredoxin component of ring-hydroxylating dioxygenase	*Paraburkholderia xenovorans* LB400[3]
ditK	ABE36491.1	Transcriptional regulator, TetR family	*Paraburkholderia xenovorans* LB400[3]
ditM	ABE36493.1	Hydrolase	*Paraburkholderia xenovorans* LB400[3]
ditN	ABE36494.1	3-hydroxyacyl CoA dehydrogenase	*Paraburkholderia xenovorans* LB400[3]
ditO	ABE36495.1	Thiolase	*Paraburkholderia xenovorans* LB400[3]
ditP	ABE36496.1	Conserved hypothetical protein	*Paraburkholderia xenovorans* LB400[3]
ditQ	ABE36497.1	Cytochrome P450	*Paraburkholderia xenovorans* LB400[3]
ditU	ABE36529.1	Cytochrome P450	*Paraburkholderia xenovorans* LB400[3]

Table S7.Genomic characteristics of *P. abieticivorans*.

Attribute	Genome
Genbank ID	GCA_023509015.1
Size (bp)	6,715,763
No. scaffolds/contigs	1
Cov. (fold)	149
G+C content (mol%)	62
Total genes	6,087
Protein-coding genes	5,987
KO numbers	3,449
tRNA	74
rRNA (5S, 16S, 23S)	8, 7, 7
Pseudogenes[a]	139

[a]The number of total pseudogenes indicated includes genes with ambiguous residues, frameshifted genes, incomplete genes, genes with internal stops or other multiple problems.

Table S8. Number of genes associated with the general COG functional categories in *P. abieticivorans*.

Code	Value	% of total	Description
J	245	4.09	Translation
A	25	0.4	RNA processing and modification
K	231	3.9	Transcription
L	238	3.97	Replication, recombination and repair
B	19	0.3	Chromatin structure and dynamics
D	72	1.2	Cell cycle control, mitosis and meiosis
Y	2	0.03	Nuclear structure
V	46	0.8	Defense mechanism
T	152	2.5	Signal transduction mechanisms
M	188	3.2	Cell wall/membrane biogenesis
N	96	1.6	Cell motility
Z	12	0.2	Cytoskeleton
W	1	0.02	Extracellular structures
U	158	2.7	Intracellular trafficking and secretion
O	203	3.4	Posttranslational modification, protein turnover, chaperones
C	258	4.3	Energy production and conversion
G	230	3.8	Carbohydrate transport and metabolism
E	270	4.5	Amino acid transport and metabolism
F	95	1.6	Nucleotide transport and metabolism
H	179	2.9	Coenzyme transport and metabolism
I	94	1.57	Lipid transport and metabolism
P	212	3.5	Inorganic transport and metabolism
Q	88	1.47	Secondary metabolites biosynthesis, transport and catabolism
R	702	11.8	General function prediction only
S	1347	22.46	Function unknown

Table S9. Phenotypic characteristics distinguishing *P. abieticivorans* from phylogenetically closely related *Pseudomonas* type strains. *P. abieticivorans* PIA16^T (1), *P. deceptionensis* M1 ^{T} (2), *P. lundensis* 573 ^{T}(3), *P. helleri* DSM 29165^T (4), *P. laurylsulfatiphila* DSM 105097^T (5). All data for *P. abieticivorans* are from this study, and references for the other data are provided by each species number indicator on the top row. All strains were grown on trypticase soy broth agar prior to fatty acid analysis. +, positive, -, negative; w, weak; tr, trace (<1%), not reported (nr).

**Characteristics**	**1**	**2**[4]	**3**[5]	**4**[5]	**5**[6]
Temperature	4-30	-4-34	4-36	4-31	nr
Hydrolysis (β-glucosidase) (esculin)	w	+	nr	nr	-
Hydrolysis (protease) (gelatin)	-	-	-	-	-
Assimilation (arabinose)	+	+	+	w	-
Assimilation (mannose)	-	+	+	w	+
Assimilation (*N*-acetylglucosamine)	-	-	-	-	+
Assimilation (phenylacetic acid)	-	-	nr	nr	+
**Enzyme**					
Alkaline phosphatase	+	-	+	nr	nr
Valine arylamidase	+	-	-	nr	nr
Naphthol-AS-BI-phosphohydrolase	+	+	-	nr	nr
Catalase	-	+	nr	+	+
**Cellular fatty acid composition**					
$C_{12:0}$	4.11	3.6	1.2	3.2	3.17
$C_{14:0}$	tr	1.7	tr	tr	nr
$C_{16:0}$	31.57	34.9	33.2	32.4	31.67
$C_{10:0}$ 3-OH	3	5.4	3.5	3.7	4.02
$C_{12:0}$ 2-OH	3.05	5.8	4.9	3.7	4.07
$C_{12:0}$ 3-OH	3.49	5.6	nr	nr	4.63
$C_{17:0}$ cyclo	12.79	16.1	1.2	17.0	1.26
Summed feature 3:$C_{16:1}$ ω7c/ω6c	26.12	21.5	37.2	21.1	38.13
Summed feature 8: $C_{18:1}$ω7c/ω6c	13.96	nr	nr	nr	nr
DNA G+C content (mol%)	61.5	58.5	58.5	58.1	60
16S rRNA sequence similarity to strain	100	97.59	97.35	97.45	99.15
rpoD sequence similarity to PIA16	100	83.57	91.25	82.66	89.00
ANI similarity to strain PIA16	100	80.88	80.8	80.9	81.93

Table S10. *Pseudomonas sp.* used for collinear analysis including strain, taxonomic identification, and NCBI assembly ID.

id	Name	Strain	Taxid	NCBI assembly id
pp1	*Pseudomonas abieticivorans*	PIA16^T	2931382	GCF_023509015.1
pr1	*Pseudomonas resinovorans*	NBRC 106553	1245471	GCF_000412695.1
pm1	*Pseudomonas multiresinivorans*		95301	GCF_012971725.1
pv1	*Pseudomonas vancouverensis*	LMG 20222^T	95300	GCF_900105825.1
pa1-54	*Pseudomonas abietaniphila*	BKME-9^T	89065	GCF_900100795.1
px1-3	*Paraburkholderia xenovorans*	LB400^T	266265	GCF_000013645.1

Table S11. Blast of *dit* cluster-encoded proteins against *Pseudomonas abieticivorans*. Hit denotes the name of the sequence found in the BLAST search and description is the Prokka-annotated description of said identified sequence. E-value is the measure of quality of the match. Higher E-values indicate that BLAST found a less homologous sequence. Identity % denotes the percentage of identical residues in the query and hit sequence.

Gene	Query NCBI accession number	PIA16 NCBI accession number	Description	E-value	Percent identity %
ditI	AAD21071.1	WP_249674695.1	Diacetyl reductase [(S)-acetoin	6.042e-152	83.83
ditA2	AAD21061.1	WP_249674693.1	Biphenyl dioxygenase subunit	5.864e-121	86.70
ditA1	AAD21063.1	WP_249674699.1	Biphenyl 2,3-dioxygenase sub	0	93.09
ditH	AAD21070.1	WP_249674700.1	putative protein YisK	0	82.57
ditG	AAD21069.1	WP_249674701.1	3-oxoacyl-[acyl-carrier-protein	2.814e-105	71.74
ditF	AAD21068.1	WP_249674704.1	hypothetical protein	0	88.06
ditR	AAD21072.1	WP_249674706.1	Bacterial transcriptional regulator	8.720e-139	72.59
ditE	AAD21067.1	WP_249674707.1	Enterobactin exporter EntS	1.388e-148	63.08
ditD	AAD21066.1	WP_249674708.1	putative protein	2.649e-144	68.71
ditC	AAD21065.1	WP_249674709.1	Iron-dependent extradiol	0	82.32
ditB	AAD21064.1	WP_249674710.1	Cyclopentanol dehydrogenase	1.769e-140	81.27
ditA3	AAD21062.1	WP_249674711.1	4Fe-4S single cluster domain	2.969e-17	81.33
ORF2	AAD21074.1	WP_249674712.1	Long-chain-fatty-acid--CoA	0	76.29
ORF3	AAR83736.1	WP_249674685.1	Crotonobetainyl-CoA reductase	0	78.53
ORF4	AAR83737.1	WP_249674686.1	Amidohydrolase	0	90.11
ditQ	AAR83738.1	WP_249674687.1	Putative cytochrome P450	0	84.47
ditP	AAR83739.1	WP_249674688.1	hypothetical protein	7.441e-69	66.87
ditO	AAR83740.1	WP_249674689.1	Beta-ketoadipyl-CoA thiolase	0	78.77
ditN	AAR83741.1	WP_249674690.1	3-hydroxybutyryl-CoA	3.404e-180	80.13
ditM	AAR83742.1	WP_249674691.1	Fumarylacetoacetate (FAA)	9.019e-179	83.04
ditL	AAR83743.1	WP_249674692.1	2-keto-4-carboxy-3-hexenedioate	0	85.80
ditK	AAR83744.1	WP_249674693.1	hypothetical protein	3.843e-127	84.95
ditU	ABE36529.1	WP_249674723.1	Putative cytochrome P450	0	64.30

Table S12. Description of Sequence Read Archive (SRA) accession numbers of each sample used for amplicon sequencing.

sample name	Libtype	Accession number
w0-s1	bv1-8 bacteria ONT amplicon library	https://www.ncbi.nlm.nih.gov/sra/SRX21853827
w0-s3	bv1-8 bacteria ONT amplicon library	https://www.ncbi.nlm.nih.gov/sra/SRX21853828
w2-s5	bv1-8 bacteria ONT amplicon library	https://www.ncbi.nlm.nih.gov/sra/SRX21853839
w2-s6	bv1-8 bacteria ONT amplicon library	https://www.ncbi.nlm.nih.gov/sra/SRX21853850
w4-s8	bv1-8 bacteria ONT amplicon library	https://www.ncbi.nlm.nih.gov/sra/SRX21853853
w4-s9	bv1-8 bacteria ONT amplicon library	https://www.ncbi.nlm.nih.gov/sra/SRX21853854
w8-s11	bv1-8 bacteria ONT amplicon library	https://www.ncbi.nlm.nih.gov/sra/SRX21853855
w8-s12	bv1-8 bacteria ONT amplicon library	https://www.ncbi.nlm.nih.gov/sra/SRX21853856
w12-s14	bv1-8 bacteria ONT amplicon library	https://www.ncbi.nlm.nih.gov/sra/SRX21853857
w12-s15	bv1-8 bacteria ONT amplicon library	https://www.ncbi.nlm.nih.gov/sra/SRX21853858
w16-s17	bv1-8 bacteria ONT amplicon library	https://www.ncbi.nlm.nih.gov/sra/SRX21853829
w16-s18	bv1-8 bacteria ONT amplicon library	https://www.ncbi.nlm.nih.gov/sra/SRX21853830
w20-s20	bv1-8 bacteria ONT amplicon library	https://www.ncbi.nlm.nih.gov/sra/SRX21853831
w20-s21	bv1-8 bacteria ONT amplicon library	https://www.ncbi.nlm.nih.gov/sra/SRX21853832
w24-s23	bv1-8 bacteria ONT amplicon library	https://www.ncbi.nlm.nih.gov/sra/SRX21853833
w24-s24	bv1-8 bacteria ONT amplicon library	https://www.ncbi.nlm.nih.gov/sra/SRX21853834
w0-s1	fITS2-C fungi ONT amplicon library	https://www.ncbi.nlm.nih.gov/sra/SRX21853835
w0-s3	fITS2-C fungi ONT amplicon library	https://www.ncbi.nlm.nih.gov/sra/SRX21853836
w2-s5	fITS2-C fungi ONT amplicon library	https://www.ncbi.nlm.nih.gov/sra/SRX21853837
w2-s6	fITS2-C fungi ONT amplicon library	https://www.ncbi.nlm.nih.gov/sra/SRX21853838
w4-s8	fITS2-C fungi ONT amplicon library	https://www.ncbi.nlm.nih.gov/sra/SRX21853840
w4-s9	fITS2-C fungi ONT amplicon library	https://www.ncbi.nlm.nih.gov/sra/SRX21853841
w8-s11	fITS2-C fungi ONT amplicon library	https://www.ncbi.nlm.nih.gov/sra/SRX21853842
w8-s12	fITS2-C fungi ONT amplicon library	https://www.ncbi.nlm.nih.gov/sra/SRX21853843
w12-s14	fITS2-C fungi ONT amplicon library	https://www.ncbi.nlm.nih.gov/sra/SRX21853844
w12-s15	fITS2-C fungi ONT amplicon library	https://www.ncbi.nlm.nih.gov/sra/SRX21853845
w16-s17	fITS2-C fungi ONT amplicon library	https://www.ncbi.nlm.nih.gov/sra/SRX21853846
w16-s18	fITS2-C fungi ONT amplicon library	https://www.ncbi.nlm.nih.gov/sra/SRX21853847
w20-s20	fITS2-C fungi ONT amplicon library	https://www.ncbi.nlm.nih.gov/sra/SRX21853848
w20-s21	fITS2-C fungi ONT amplicon library	https://www.ncbi.nlm.nih.gov/sra/SRX21853849
w24-s23	fITS2-C fungi ONT amplicon library	https://www.ncbi.nlm.nih.gov/sra/SRX21853851
w24-s24	fITS2-C fungi ONT amplicon library	https://www.ncbi.nlm.nih.gov/sra/SRX21853852

Table S13. Metagenome-assembled genomes (MAGs) NCBI accession numbers from https://www.ncbi.nlm.nih.gov/sra/SRX18691017 and https://www.ncbi.nlm.nih.gov/sra/SRX18691016.

MAG ID	Biosample ID	GenBank ID	Link
Bin1	SAMN32241433	GCA_028283825.1	https://www.ncbi.nlm.nih.gov/nuccore/JAPWJW000000000
Bin2	SAMN32241434	GCA_027498035.1	https://www.ncbi.nlm.nih.gov/nuccore/2418877976
Bin3	SAMN32241435	GCA_028283855.1	https://www.ncbi.nlm.nih.gov/nuccore/JAPWJX000000000
Bin4	SAMN32241436	GCA_028283765.1	https://www.ncbi.nlm.nih.gov/nuccore/JAPWJY000000000
Bin5	SAMN32241437	GCA_028283745.1	https://www.ncbi.nlm.nih.gov/nuccore/JAPWJZ000000000
Bin6	SAMN32241438	GCA_028283795.1	https://www.ncbi.nlm.nih.gov/nuccore/JAPWKA000000000
Bin7	SAMN32241439	GCA_028283785.1	https://www.ncbi.nlm.nih.gov/nuccore/JAPWKB000000000
Bin8	SAMN32241440	GCA_028283725.1	https://www.ncbi.nlm.nih.gov/nuccore/JAPWKC000000000
Bin9	SAMN32241441	GCA_028283705.1	https://www.ncbi.nlm.nih.gov/nuccore/JAPWKD000000000
Bin10	SAMN32241442	GCA_028283655.1	https://www.ncbi.nlm.nih.gov/nuccore/JAPWKE000000000
Bin11	SAMN32241443	GCA_028283685.1	https://www.ncbi.nlm.nih.gov/nuccore/JAPWKF000000000
Bin12	SAMN32241444	GCA_028283625.1	https://www.ncbi.nlm.nih.gov/nuccore/JAPWKG000000000
Bin13	SAMN32241445	GCA_028283605.1	https://www.ncbi.nlm.nih.gov/nuccore/JAPWKH000000000
Bin14	SAMN32241446	GCA_028283645.1	https://www.ncbi.nlm.nih.gov/nuccore/JAPWKI000000000
Bin15	SAMN32241447	GCA_028283575.1	https://www.ncbi.nlm.nih.gov/nuccore/JAPWKJ000000000

Supplementary figures

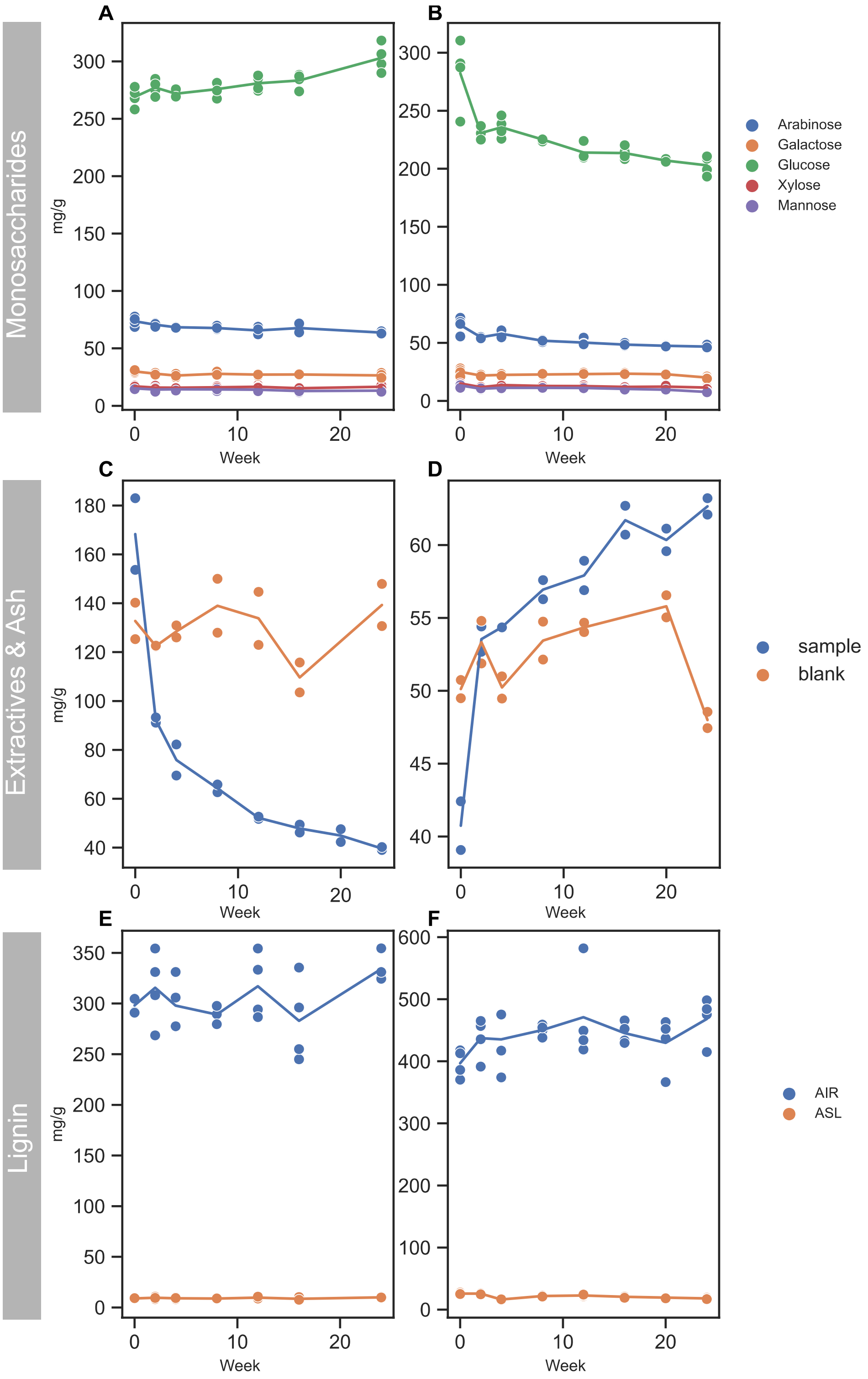

Figure S1. Chemical analyses of lignin, carbohydrates, and extractives from spruce bark after incubation over time. Monosaccharide composition after sulfuric acid hydrolysis for A) uninoculated control, and B) biotic sample. Total acetone extract C) and D) ash content. Acid insoluble residue (AIR) and acid soluble lignin (ASL) for E) uninoculated control, and F) biotic sample. Mean and standard deviations are based upon duplicate biological experiments and two technical replicates except extractive and ash measurements which are based on biological triplicate experiments. Differences in absolute values between the blank and sample timepoint zero stem from a different batch of bark being used for the blank samples. Source data are provided as a Source Data file.

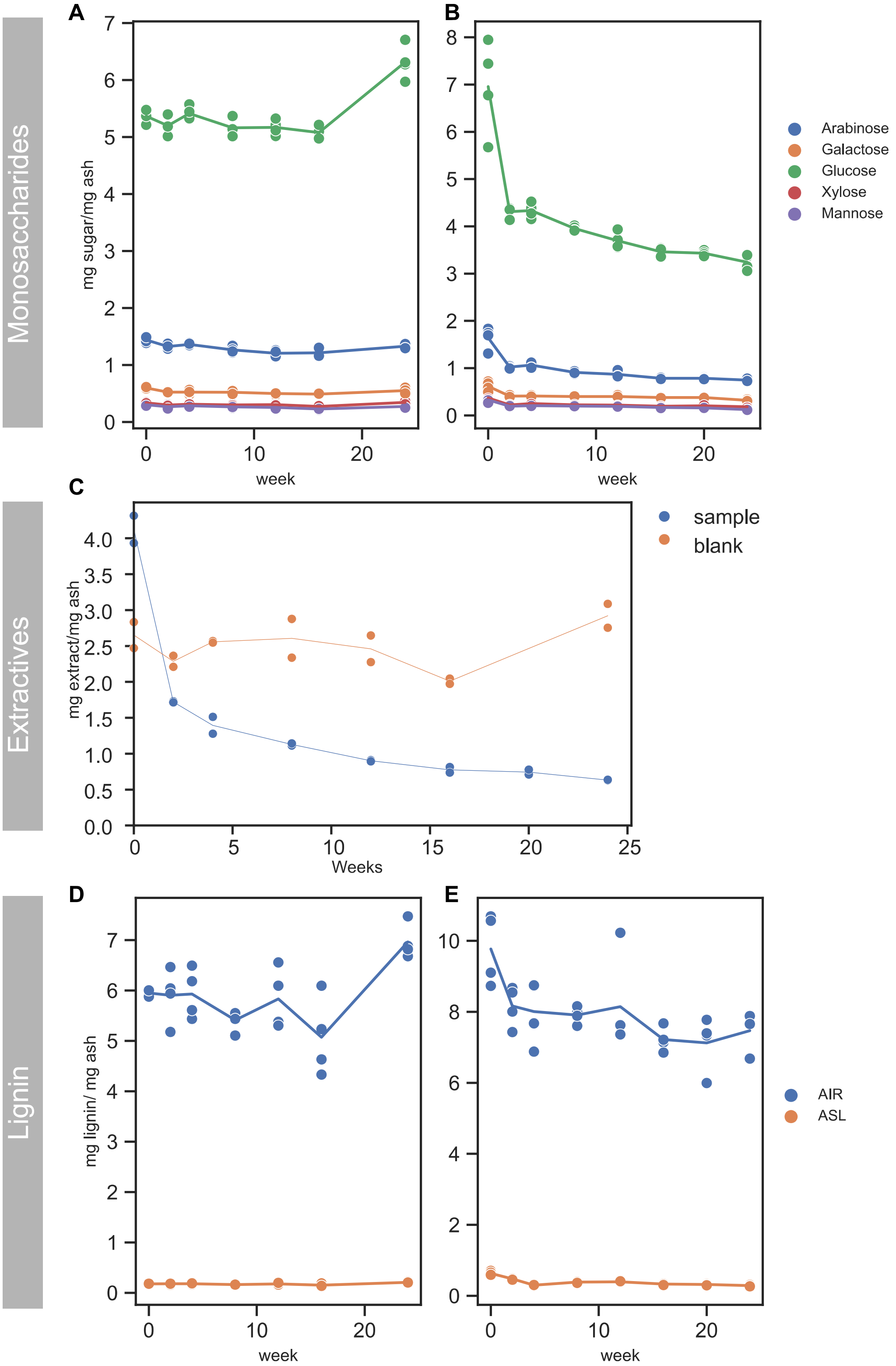

Figure S2. Chemical analyses as in Fig. S2, normalized against the ash content. Monosaccharide composition after sulfuric acid hydrolysis in the A) uninoculated control and B) biotic sample. C) Total acetone extract. Acid insoluble residue (AIR) and acid soluble lignin (ASL) in the D) uninoculated control and E) biotic sample. Mean and standard deviation are based upon duplicate biological experiments and two technical replicates for all experiments except extractive measurements which are based on biological triplicate experiments. Differences in absolute values between the blank and sample timepoint zero stem from a different batch of bark being used for the blank samples. Source data are provided as a Source Data file.

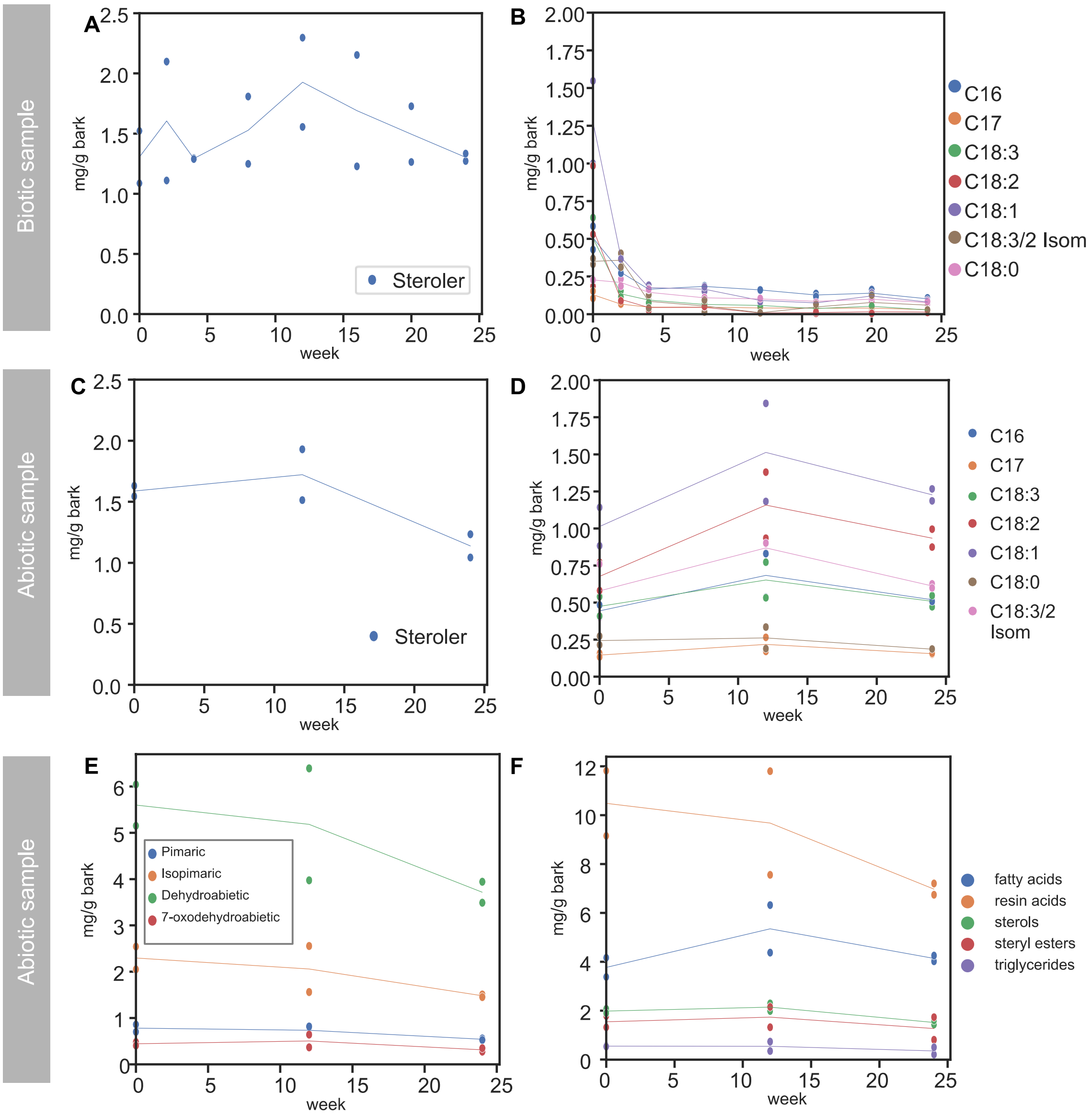

Figure S3. Changes in the extractive groups and compounds in the biotic and the abiotic (blank) sample. A) Unidentified sterol at RT=24.33 in the biotic sample. B) Fatty acids in the biotic sample. C) Unidentified sterol at RT=24.33 in the abiotic sample. D) Fatty acids in the abiotic sample. E) Resin acids in the abiotic sample. F) Extractive groups in the abiotic sample (blank). Mean and standard deviations are based upon duplicate biological experiments. Differences in absolute values between the blank and sample timepoint zero stem from a different batch of bark being used for the blank samples. Source data are provided as a Source Data file.

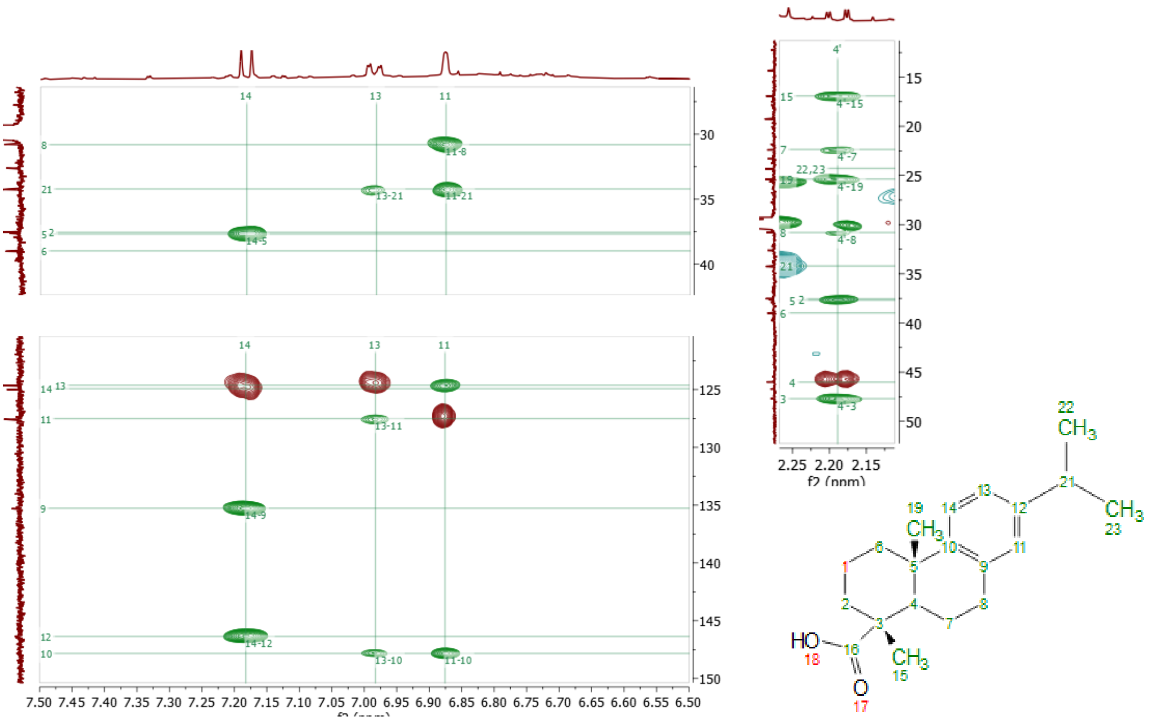

Figure S4. Structure elucidation of dehydroabietic acid in spruce bark extract from the two-week degraded sample using 2D-NMR. Important cross-signals in HSQC (red) and HMBC (green) aromatic region with assignment of dehydroabietic acid. The proton 4 showed several correlations confirming this structure, including the correlations to carbons 2, 3, 7, 8, 15, 16, and 19. Methyl protons on 22/23 show clear correlations to the aromatic carbon 12 whereas the methyl protons 19 show correlations to the aromatic carbon 10 and the methyl protons 15 have correlation to the carboxylic acid 16, confirming the presence of this group.

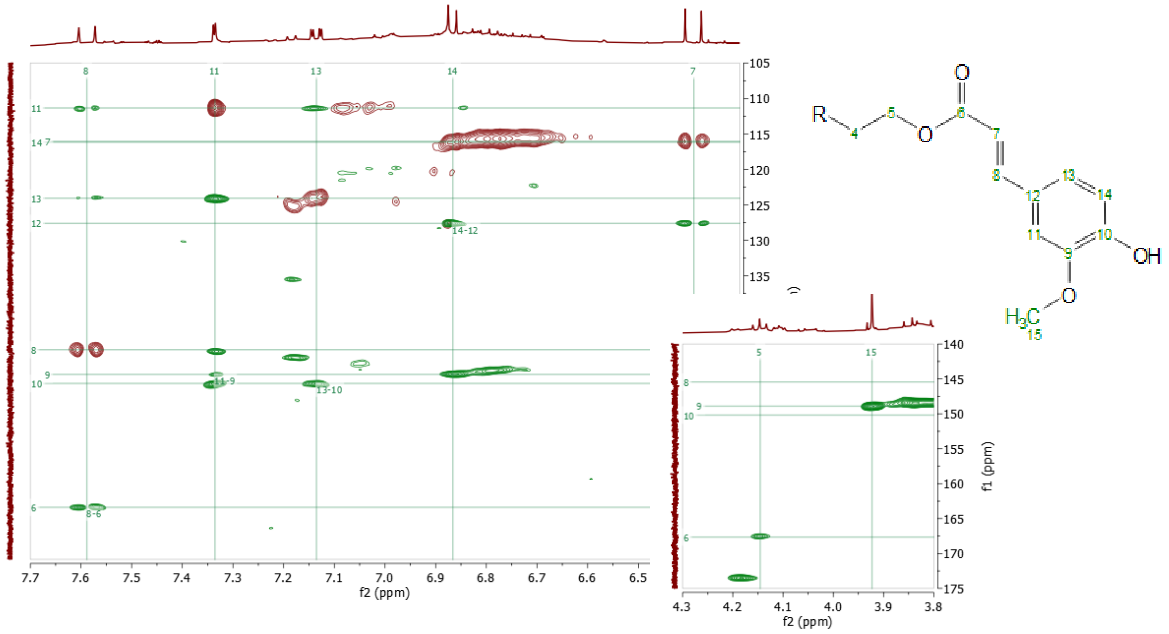

Figure S5. Structure elucidation of ferulic ester compound in spruce bark extract from the 24-week degraded sample using 2D-NMR. Important cross-signals in HSQC (red) and HMBC (green) aromatic region with assignment of a ferulic ester compound. The inset shows a correlation between aromatic ring carbon 9 to methoxy group proton 15, as well as a correlation between the ester carbon 6 towards a proton 5. Clear cross signals originating from the aromatic ring as well as cross signals originating from a double bond can be seen in the spectrum. For the double bond, the J-couplings of 16 Hz corresponded well to those obtained from a double bond in trans figuration7. From the HMBC cross signals connecting the aromatic ring and the double bond could be seen. Most notably, the proton on 8 have cross-correlations in the HMBC to the aromatic carbons 11 and 13. In addition to this, the 8 proton also has a correlation to an ester carbon 6. In the inset of the figure, a correlation between the ester carbon 6 towards a proton 5 can be seen indicating that this is a ferulic ester compound rather than a ferulic acid.

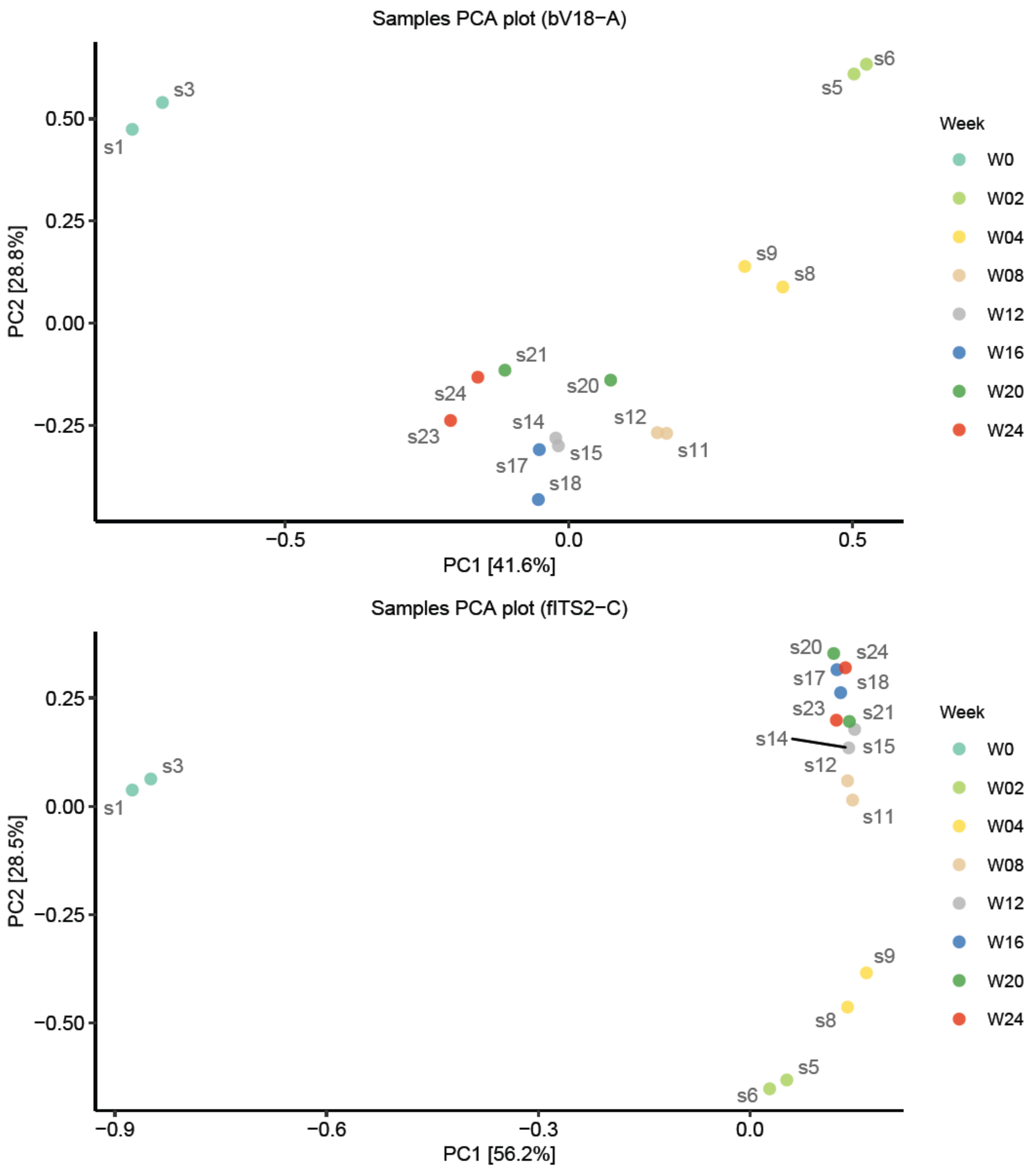

Figure S6. Principle component analysis (PCA). Identification of samples with similar microbial community was done using multivariate statistics, to illustrate sample similarities. Each point represents a community in a specific sample and is colored by the week sampled, and good agreement between biological replicates was confirmed by principal component analysis (PCA) A) bacteria B) fungi.

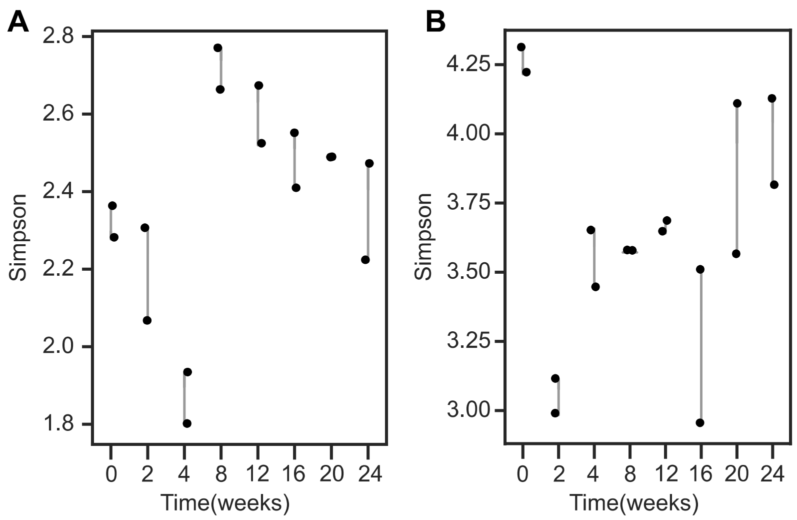

Figure S7. Alpha-diversity index of microorganisms growing on spruce bark over time. For A) Fungi B) Bacteria. Individual data points are shown and data points from the same week linked by a line for clarity. Source data are provided as a Source Data file.

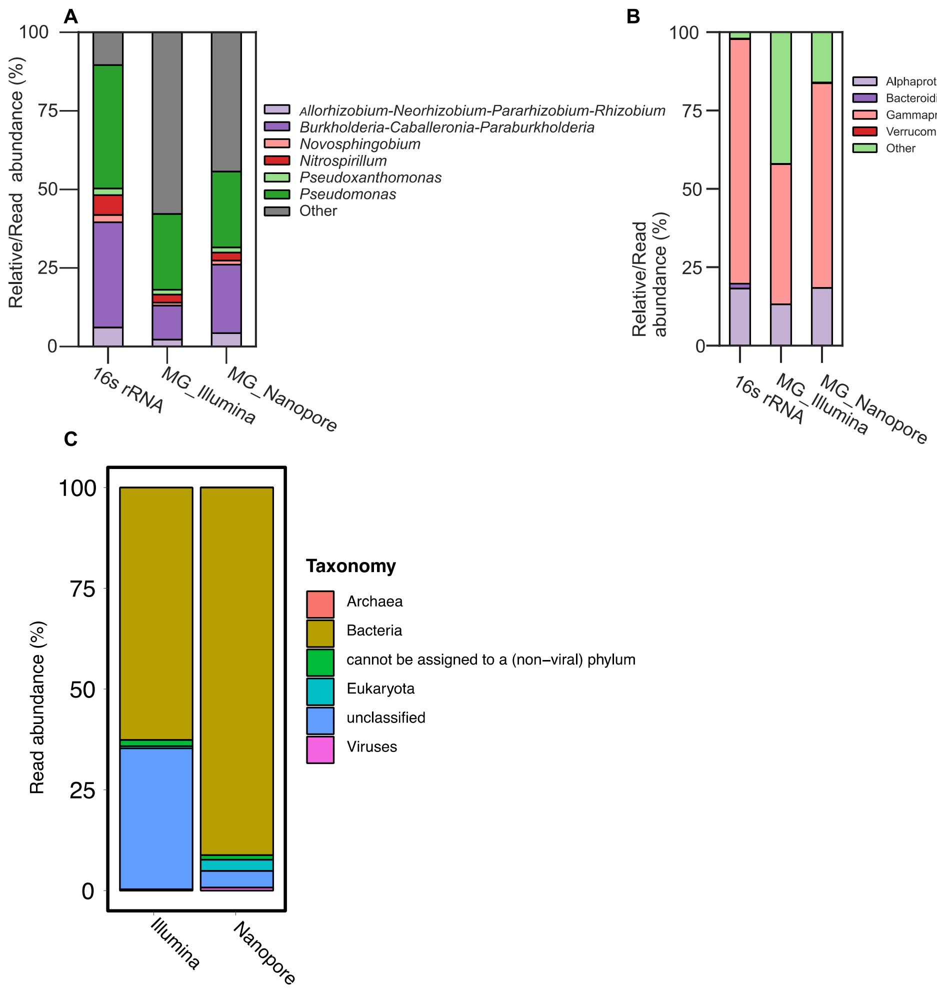

Figure S8. Microbial taxonomic composition after two weeks of spruce bark degradation. A) Relative genus, B) class abundances based on 16S rRNA gene target sequencing (16S) and whole metagenome (MG) reads both Illumina and Nanopore. Comparison of fungal, bacterial and unclassified abundance in sample based on C) reads. Source data are provided as a Source Data file.

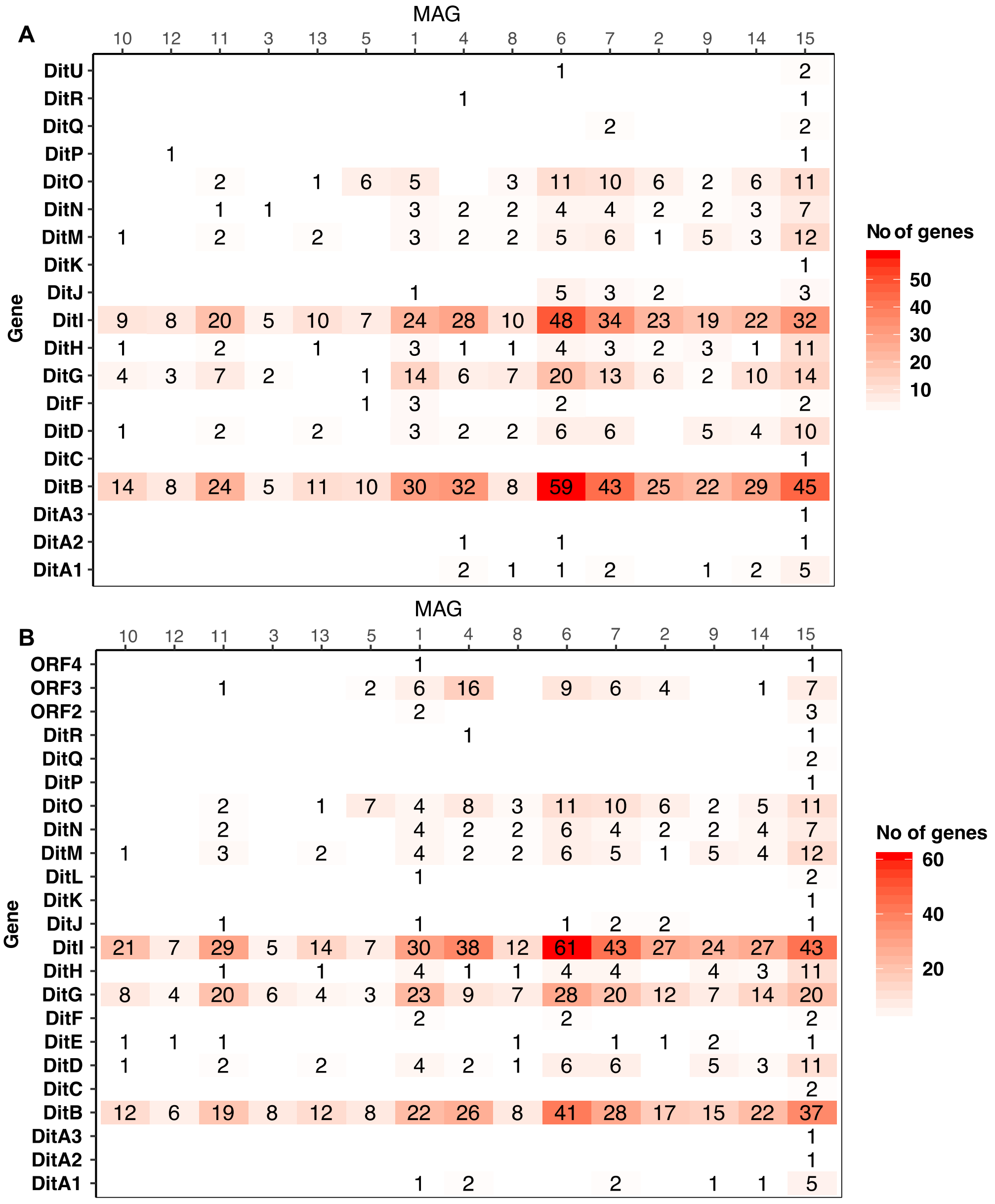

Figure S9. Abundance of *dit* cluster protein sequences in MAGs. BLAST analysis of MAGs derived from two-week sample of degraded spruce bark. Using either sequences from A) *Paraburkholderia xenovorans* LB400^{T} or B) *Pseudomonas abietaniphila* BKME-9^{T} as a query sequence.

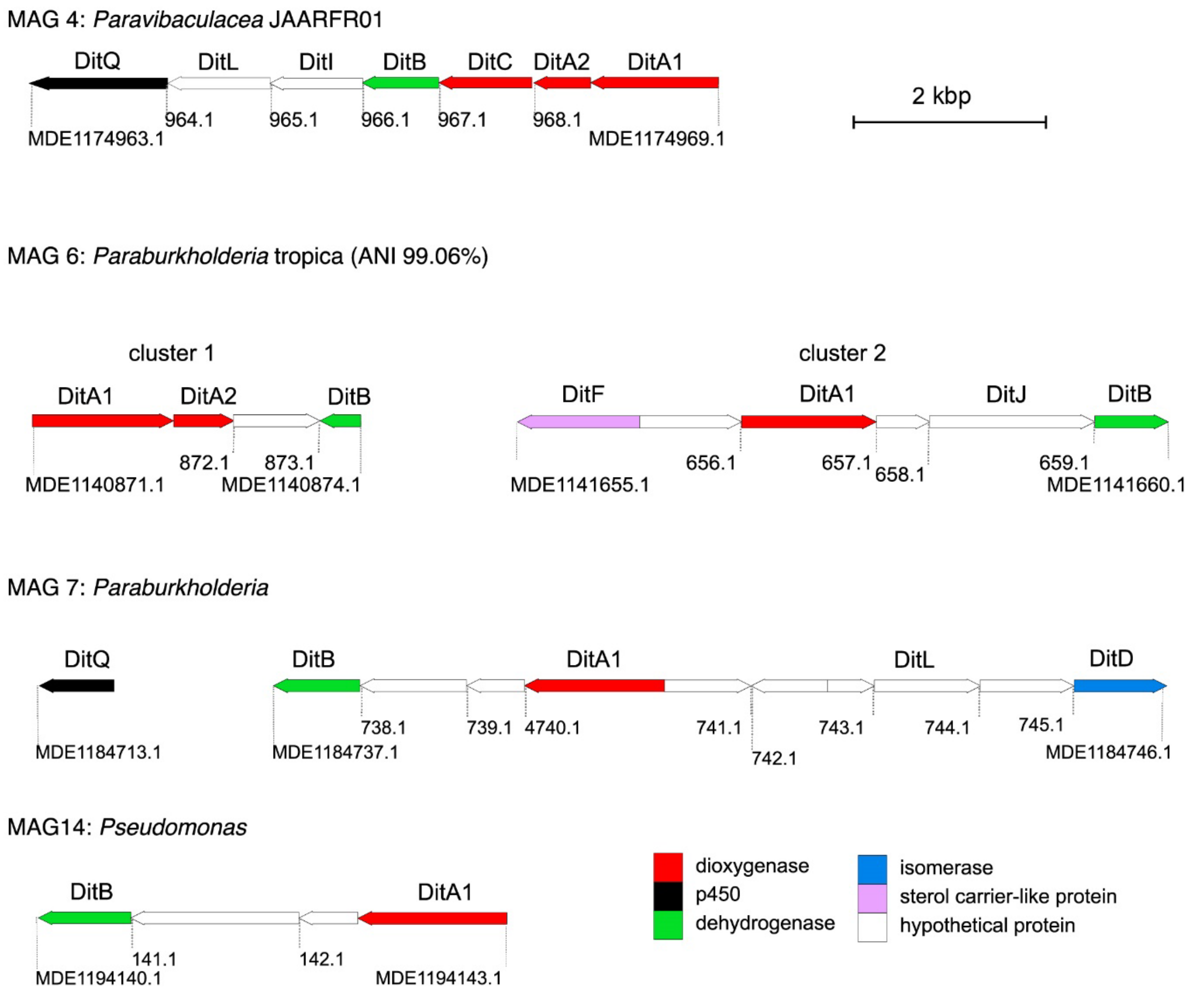

Figure S10. Genetic organization of putative *dit* gene clusters, or *dit*-cluster-like fragments, in the MAGs. Genes encoding proteins of predicted functions (BLAST) are color coded, and locus tags are indicated below the start and end of each gene cluster.

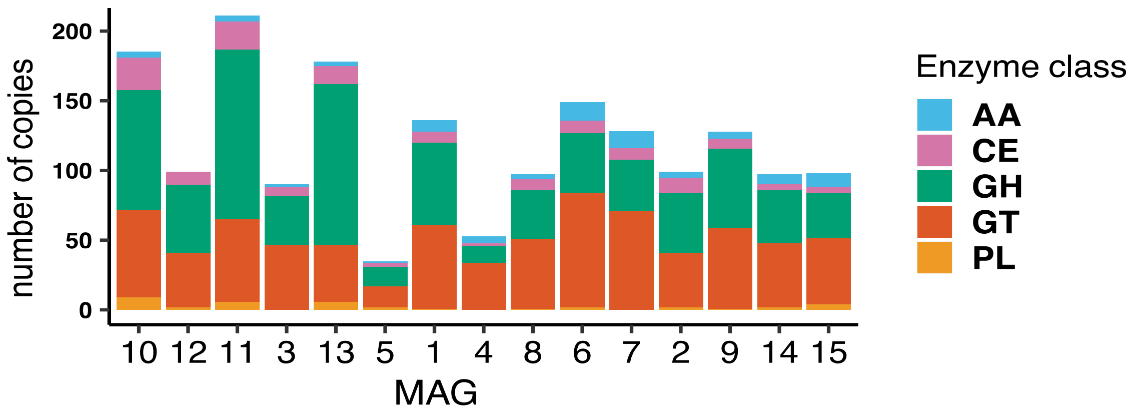

Figure S11. CAZyme analysis of the MAGs. The different CAZy classes are color coded, with auxiliary activity (AA) in blue, carbohydrate esterase (CE) in purple, glycoside hydrolase (GH) in green, glycosyltransferase (GT) in red, and polysaccharide lyase (PL) in orange.

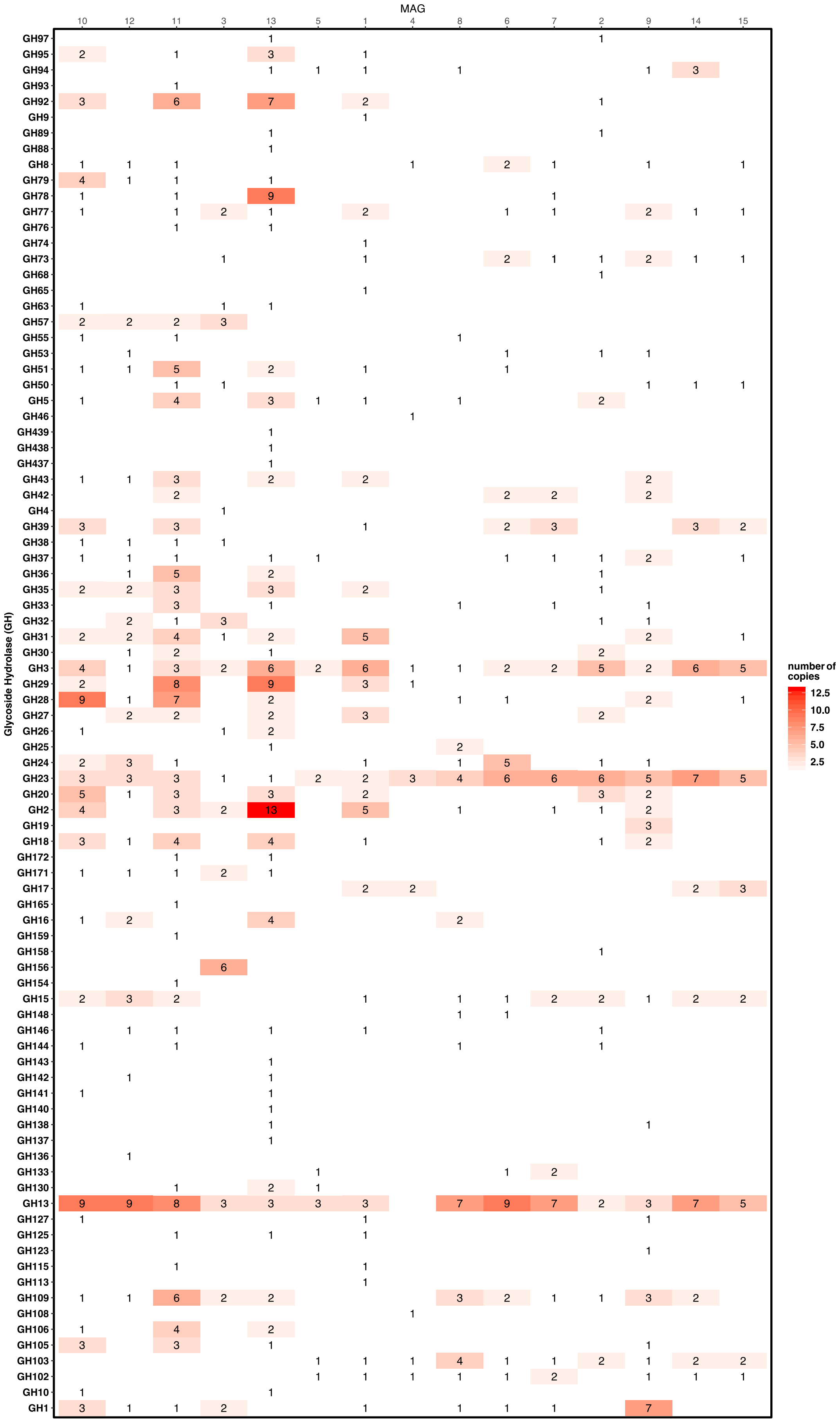
MAG
10 12 11 3 13 5 1 4 8 6 7 2 9 14 15
Glycoside Hydrolase (GH)
GH97
GH95
GH94
GH93
GH92
GH9
GH89
GH88
GH8
GH79
GH78
GH77
GH76
GH74
GH73
GH68
GH65
GH63
GH57
GH55
GH53
GH51
GH50
GH5
GH46
GH439
GH438
GH437
GH43
GH42
GH4
GH39
GH38
GH37
GH36
GH35
GH33
GH32
GH31
GH30
GH3
GH29
GH28
GH27
GH26
GH25
GH24
GH23
GH20
GH2
GH19
GH18
GH172
GH171
GH17
GH165
GH16
GH159
GH158
GH156
GH154
GH15
GH148
GH146
GH144
GH143
GH142
GH141
GH140
GH138
GH137
GH136
GH133
GH130
GH13
GH127
GH125
GH123
GH115
GH113
GH109
GH108
GH106
GH105
GH103
GH102
GH10
GH1
number of copies
12.5
10.0
7.5
5.0
2.5

Figure S12. Number of predicted copies and CAZy family membership of MAG glycoside hydrolases (GH).

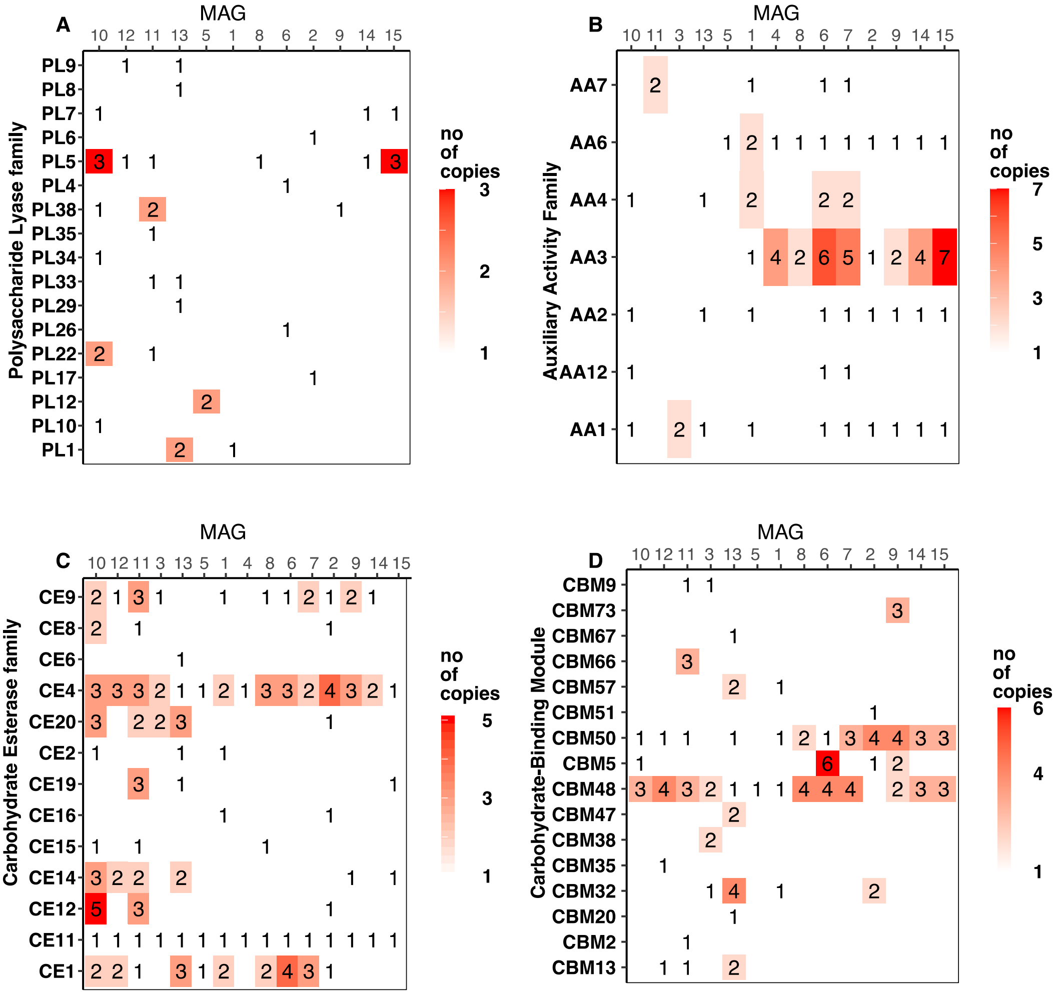

Figure S13. Number of predicted copies and CAZy family membership in the MAGs for A) polysaccharide lyases, B) auxiliary activities, C) carbohydrate esterases, and D) carbohydrate binding modules (CBMs).

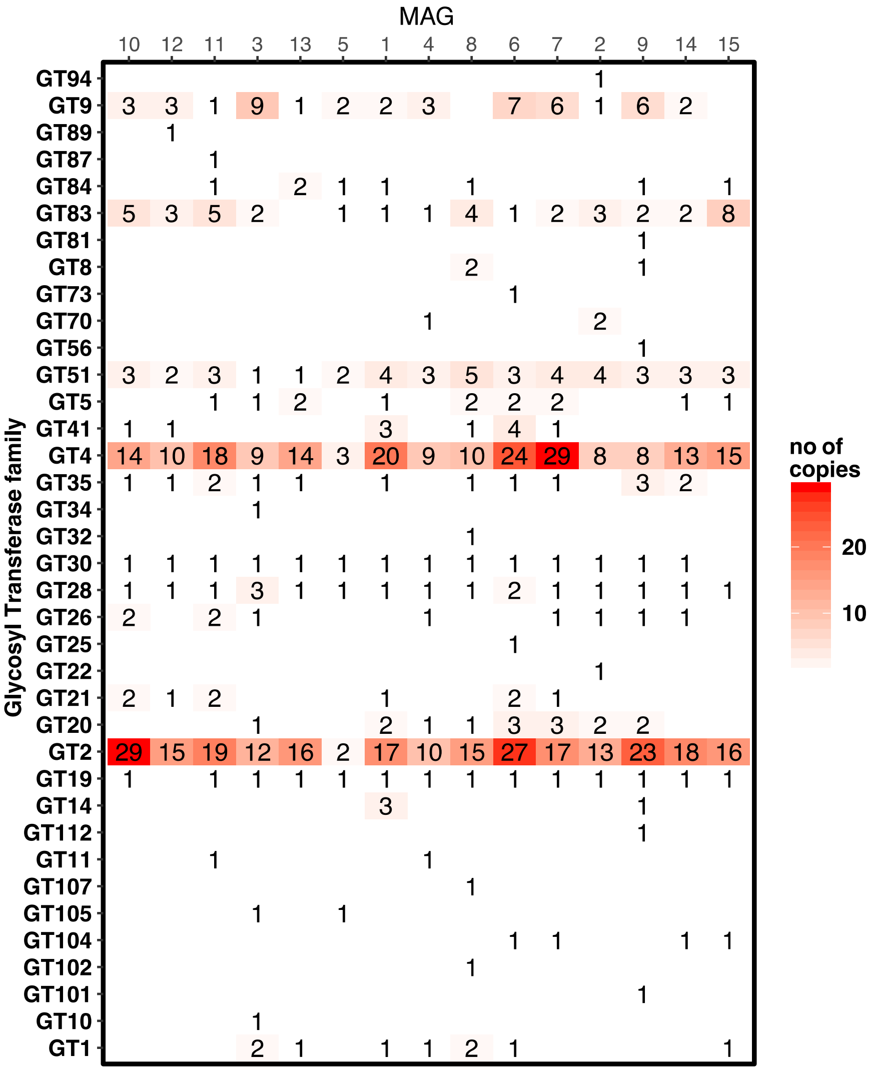

Figure S14. Number of predicted copies and CAZy family membership of MAG glycosyltransferases (GTs).

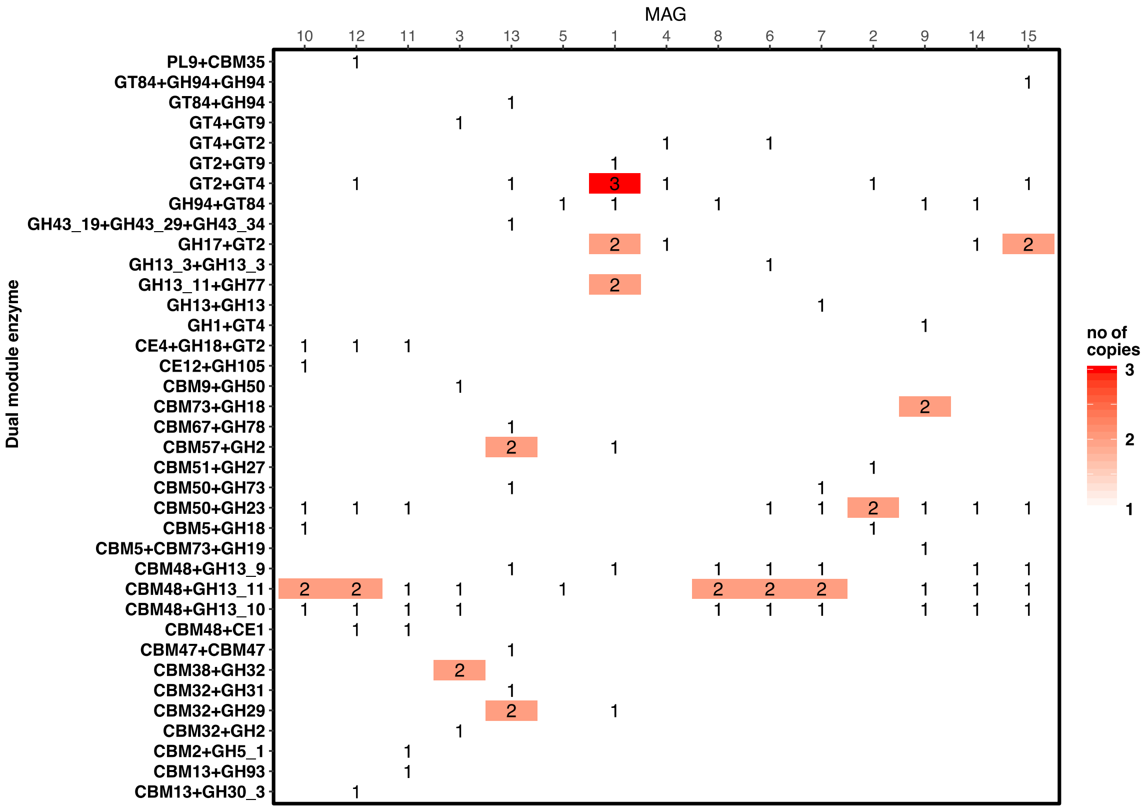

Figure S15. Multimodular CAZymes identified in the MAGs, and their domain order.

Group	Expected activity	Family	10	12	11	3	13	5	1	4	8	6	7	2	9	14	15
Algal/Proteoglycans	hyaluronate lyase	**PL8**					1										
	alginate lyase	**PL7_1**														1	
	alginate lyase	**PL7**	1														1
	alginate lyase	**PL6_1**												1			
	alginate lyase	**PL5_1**														1	2
	alginate lyase	**PL5**	3	1	1						1						1
	endo-β-1,4-glucuronan lyase	**PL38**	1		2										1		
	chondroitin lyase/chondroitinase	**PL35**			1												
	alginate lyase	**PL34**	1														
	hyaluronan,chondroitin sulfate	**PL33_1**			1		1										
	heparin-sulfate lyase	**PL12**						2									
	unsaturated glucuronyl hydrolase	**GH88**					1										
	β-glucuronidase	**GH79**	4	1	1		1										
	laminarinase	**GH55**	1		1												
	β-agarase	**GH50**			1										1	1	1
	sialidase	**GH33**			3		1				1		1		1		
	sialidase	**GH30_1**					1							1			
	laminarinase	**GH17+GT2**							2	1						1	2
	laminarinase	**GH17**								1						1	1
	exosialidase	**GH156**				6											
	α-N-acetylgalactosaminidase	**GH109**	1	1	6	2	2				3	1	1	1	3	2	
	rhamnogalacturonyl hydrolase	**GH105**	2		3		1								1		
	acetyl esterase	**CE9**	2	1	3	1			1		1	1	2	1	2	1	
	β-agarase	**CBM9+GH50**				1											
beta–glycosidase	β-galactosidases	**GH42**			2							2	2		2		
	β-xylosidase	**GH39**	3		3				1			4	1			3	2
	β-galactosidases	**GH35**	2	2	3		3		2					1			
	β-glycosidase	**GH3**	4	1	3	2	6	2	6	1	1	2	2	5	2	6	5
	β-glycosidase	**GH2**	4		3	1	11		4		1		1	1	2		
	β-galactosidase	**GH165**			1												
	β-glucosidases/β-galactosidases	**GH1+GT4**													1		
	β-glucosidases/β-galactosidases	**GH1**	3	1	1	2			1		1	1	1		6		
	β-gal	**CBM57+GH2**					2		1								
	β-gal	**CBM32+GH2**				1											
Cellulose	endo-glucanases	**GH9**							1								
	β-endo	**GH8**	1	1	1					1		2	1		1		1
	endo-β-1,4-glucanase	**GH5_5**									1			2			
	no known	**GH5_39**	1														
	endo-β-1,4-glucanase	**GH5_25**									1						
	endo-β-1,4-glucanase	**GH5_2**					1										
	endoglucanase	**GH5**			2		1		1								
	cellulase	**CBM2+GH5_1**			1												
Chitin	α-N-acetylglucosaminidase	**GH89**					1							1			
	N-acetylglucosaminidase	**GH73**				1			1			2	1	1	1	1	1
	chitosanase	**GH46**								1							
	β-N-acetylhexosaminidase	**GH20**	5	1	3		3		2					3	2		
	chitinase/lysozyme	**GH19**													2		
	chitinase	**GH18**	1		3		4		1								
	chitinase/esterase	**CE4+GH18+GT2**	1	1	1												
	acetyl esterase	**CE4**	2	2	2	2	1	1	2	1	3	3	2	4	3	2	1
	N-acetylglucosamine deacetylase	**CE14**	3	2	2		2								1		1
	chitinase	**CBM73+GH18**													2		
	chitinase	**CBM5+GH18**	1											1			
	chitinase/lysozyme	**CBM5+CBM73+GH19**													1		

(Column headings 10–15: MAG)

Category	Enzyme	Family	MAG 10	12	11	3	13	5	1	4	8	6	7	2	9	14	15
Lignin	manganese peroxidase	**AA2**	1				1		1			1	1	1	1	1	1
Lignin	Laccase	**AA1**	1			2	1		1			1	1	1	1	1	1
Mannan	α-galactosidase	**GH36**		1	5		2							1			
Mannan	α-galactosidase	**GH27**		2	2		2		3					1			
Mannan	β-mannanase	**GH26**	1			1	2										
Mannan	β-mannoside phosphorylase	**GH130**			1		2	1									
Mannan	exo-α-1,6-mannosidase	**GH125**			1		1		1								
Mannan	β-mannanase	**GH113**							1								
Mannan	α-galactosidase	**CBM51+GH27**												1			
Microbial glycan	α-mannosidases	**GH92**	3		6		7		2					1			
Microbial glycan	endo-α-1,6-mannanase	**GH76**			1		3										
Microbial glycan	α-mannosidase	**GH38**	1	1	1	1											
Microbial glycan	endo-β-1,3-glucanase	**GH30_3**			1									1			
Microbial glycan	lysozyme	**GH25**					1				2						
Microbial glycan	lysozyme	**GH24**	2	3	1				1		1	5		1	1		
Microbial glycan	peptidoglycan lytic transglycosylase	**GH23**	2	2	2	1	1	2	2	3	4	5	5	5	4	6	4
Microbial glycan	peptidoglycan β-N-acetylmuramidase	**GH171**	1	1	1	2	1										
Microbial glycan	endo-β-1,3-1,4-glycanase	**GH16_5**									1						
Microbial glycan	endo-β-1,3-glucanase	**GH16_3**	1	1			2										
Microbial glycan	β-1,3-glucanase	**GH16_21**									1						
Microbial glycan	β-1,3-glucanase	**GH16**		1			2										
Microbial glycan	endo-β-1,3-glucanase	**GH158**												1			
Microbial glycan	endo-β-1,2-glucanase	**GH144**	1		1						1			1			
Microbial glycan	peptidoglycan	**GH108**								1							
Microbial glycan	peptidoglycan lytic transglycosylase	**GH103**						1	1	1	4	1	1	2	1	2	2
Microbial glycan	peptidoglycan lytic transglycosylase	**GH102**						1	1	1	1	1	2		1	1	1
Microbial glycan	peptidoglycan hydrolase	**CBM50+GH73**					1						1				
Microbial glycan	lytic transglycosylase	**CBM50+GH23**	1	1	1							1	1	2	1	1	1
Pectin	pectate lyase	**PL9+CBM35**		1													
Pectin	pectate lyase	**PL9**					1										
Pectin	rhamnogalacturonan endolyase	**PL4**										1					
Pectin	rhamnogalacturonan endolyase	**PL26**										1					
Pectin	pectate lyase	**PL10_1**	1														
Pectin	pectate lyase	**PL1_2**					2		1								
Pectin	α-L-rhamnosidases	**GH78**	1		1		8						1				
Pectin	α-L-rhamnosidases	**GH53**		1								1		1	1		
Pectin	exo-α-1,5-L-arabinofuranosidase	**GH43_5**							1								
Pectin	exo-β-1,3-galactanase	**GH43_24**		1			1										
Pectin	endo-arabinases	**GH43**	1		1												
Pectin	α-fucosidase	**GH29**	2		8		7		2	1							
Pectin	polygalacturonase	**GH28**	9	1	7		2				1	1			2		1
Pectin	α-L-arabino/β-D-galactofuranosidase	**GH154**			1												
Pectin	β-1,2-apiosidase	**GH140**					1										
Pectin	rhamnogalacturonan α-1,2-galacturonohydrolase	**GH138**					1										
Pectin	β-1,2-L-arabinofuranosidas	**GH127**	1						1						1		
Pectin	α−L−rhamnosidase	**GH106**	1		4		2										
Pectin	pectin methylesterase	**CE8**	2		1									1			
Pectin	pectin methylesterase	**CE19**			3		1										1
Pectin	pectin acetylesterase	**CE12+GH105**	1														
Pectin	pectin acetylesterase	**CE12**	4		3									1			
Pectin	α-L-rhamnosidase	**CBM67+GH78**					1										
Pectin	α-fucosidase	**CBM32+GH29**					2		1								
Pectin	exo-α-L-1,5-arabinanase	**CBM13+GH93**			1												

Starch/Glycogen	α-glycosiadas	GH4				1											
Starch/Glycogen	α,α-trehalase	GH37	1	1	1		1	1				1	1	1	2		1
Starch/Glycogen	α-glucosidas	GH31	2	2	4	1	2		5						2		1
Starch/Glycogen	glucoamylase	GH15	2	3	2				1		1	1	2	2	1	2	2
Starch/Glycogen	amylo-α-1,6-glucosidase	GH133						1					1				
Starch/Glycogen	α-glucosidase	GH13+GH13										1	4				
Starch/Glycogen	α-amylase	GH13_7			1												
Starch/Glycogen	trehalose synthase	GH13_33														1	1
Starch/Glycogen	α-1,4-glucan/phosphate α-maltosyltransferase	GH13_3	1	1												1	1
Starch/Glycogen	α-glucosidase	GH13_26	1	1	1	1					1	1	1		1	1	1
Starch/Glycogen	α-glucosidase	GH13_23	1	1					1		2						
Starch/Glycogen	sucrose phosphorylase	GH13_18													1		
Starch/Glycogen	trehalose synthase	GH13_16	1	1	1							1	1			1	1
Starch/Glycogen	isoamylase	GH13_11+GH77							2								
Starch/Glycogen	α-glucoside linkages	GH13	2	2	3		2	2						2			
Starch/Glycogen	glycogen branching enzyme	CBM48+GH13_9					1		1		1	1	1			1	1
Starch/Glycogen	isoamylase	CBM48+GH13_11	2	2	1	1		1			2	2	2		1	1	1
Starch/Glycogen	α-amylase	CBM48+GH13_10	1	1	1						1	1	1		1	1	1
Xylan	xylanase	GH30			1												
Xylan	α-(4-O-methyl)-glucuronidase	GH115			1				1								
Xylan	xylanase	GH10	1				1										
Xylan	acetyl xylan esterase	CE6					1										
Xylan	acetyl xylan esterase	CE2	1				1		1								
Xylan	4-O-methyl-glucuronoyl methylesterase	CE15	1		1						1						
Xylan	acetyl xylan esterase	CE1	2	1			3	1	2		2	4	3	1			
Xylan	endo-β-1,6-glucanase	CBM13+GH30_3		1													
Xylan/pectin	α-L-arabinofuranosidase	GH51	1	1	5		2		1			1					
Xylan/pectin	β-D-galacto/α-L-arabinofuranosidase	GH5_13			1		1	1									
Xylan/pectin	α-L-arabinofuranosidase	GH43_9							1								
Xylan/pectin	β-D-galactofuranosidase/endo-α-1,5-L-arabinanase	GH43_37					1										
Xylan/pectin	β-D-galacto/α-L-arabinofuranosidase	GH43_34			1												
Xylan/pectin	exo-α-1,5-L-arabino/α-L-arabinofuranosidase	GH43_26			1										1		
Xylan/pectin	β-D-galacto/α-L-arabinofuranosidase	GH43_19+GH43_29+GH43_34					1										
Xylan/pectin	α-L-arabinofuranosidase	GH43_19					1										
Xylan/pectin	α-L-arabinofuranosidase	GH43_18					1										
Xylan/pectin	β-1,4-xylosidase	GH43_11													1		
Xylan/pectin	α-L-arabinofuranosidase β-D-galactofuranosidase	GH159			1												
Xylan/pectin	β-L-arabinofuranosidase	GH146		1	1		1		1					1			
Xylan/pectin	β-L-arabinofuranosidase	GH142		1			1										
Xylan/pectin	β-L-arabinofuranosidase	GH137					1										
Xyloglucan	α-L-fucosidase	GH95	2		1		3		1								
Xyloglucan	endo-xyloglucanas	GH74							1								
Xyloglucan	α-xylosidase	GH31	2	2	4	1	2		5						2		
Xyloglucan	acetylesterase	CE20	3		2	2	3							1			

Figure S16. Predicted activities and possible target substrates for relevant CAZy family members identified in the MAGs. Note that some families appear in different categories, and predictions may not show all possible activities, especially from polyspecific families.

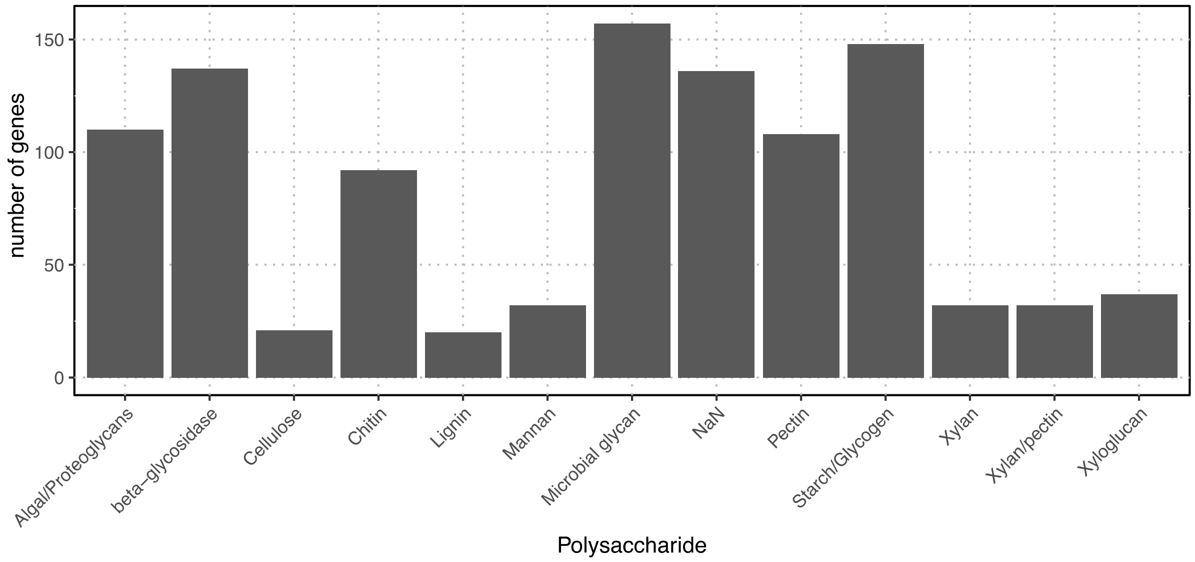

Figure S17. Number of CAZyme-encoding genes overall within the MAGs, based on the predicted activities in Figure S16.

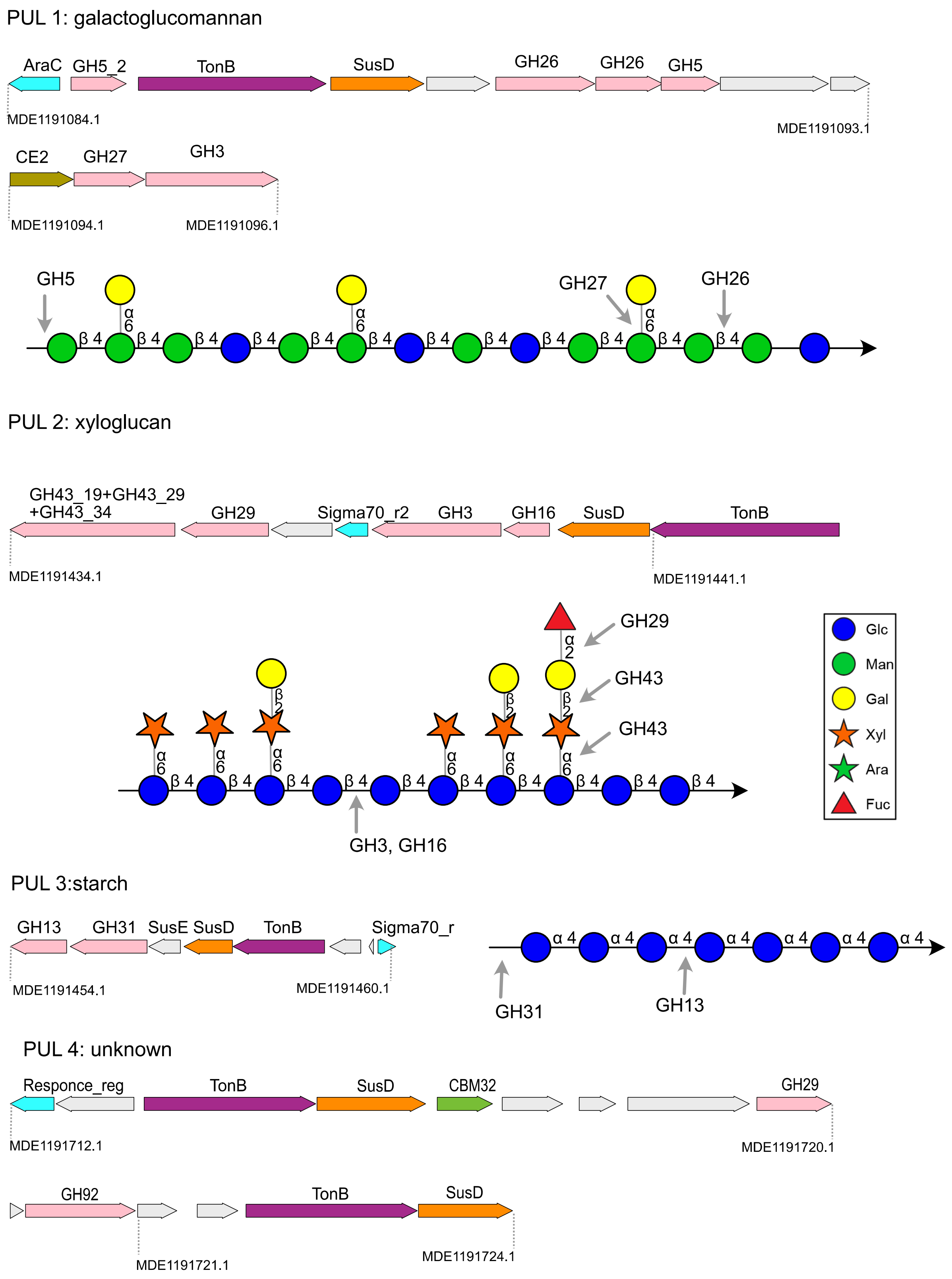

Figure S18a. Putative Polysaccharide Utilization Loci (PULs) identified in MAG13. The PUL-encoded genes are colored according to putative function, and gray arrows indicate both putatively annotated proteins and proteins of unknown function. Representative polysaccharide structures are shown close to each identified PUL.

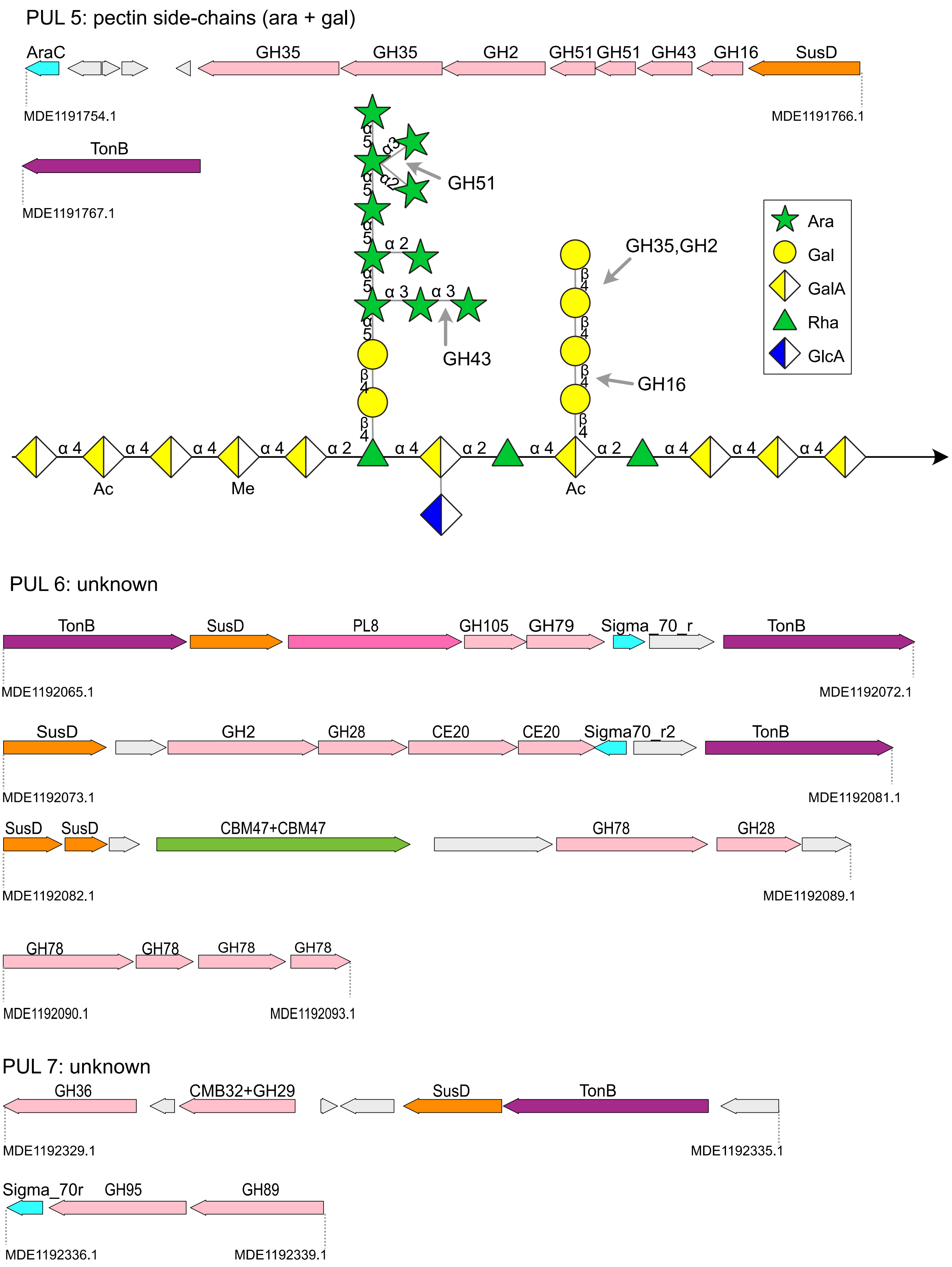

Figure S18b. Putative Polysaccharide Utilization Loci (PULs) identified in MAG13. The PUL-encoded genes are colored according to putative function, and gray arrows indicate both putatively annotated proteins and proteins of unknown function. Representative polysaccharide structures are shown close to each identified PUL.

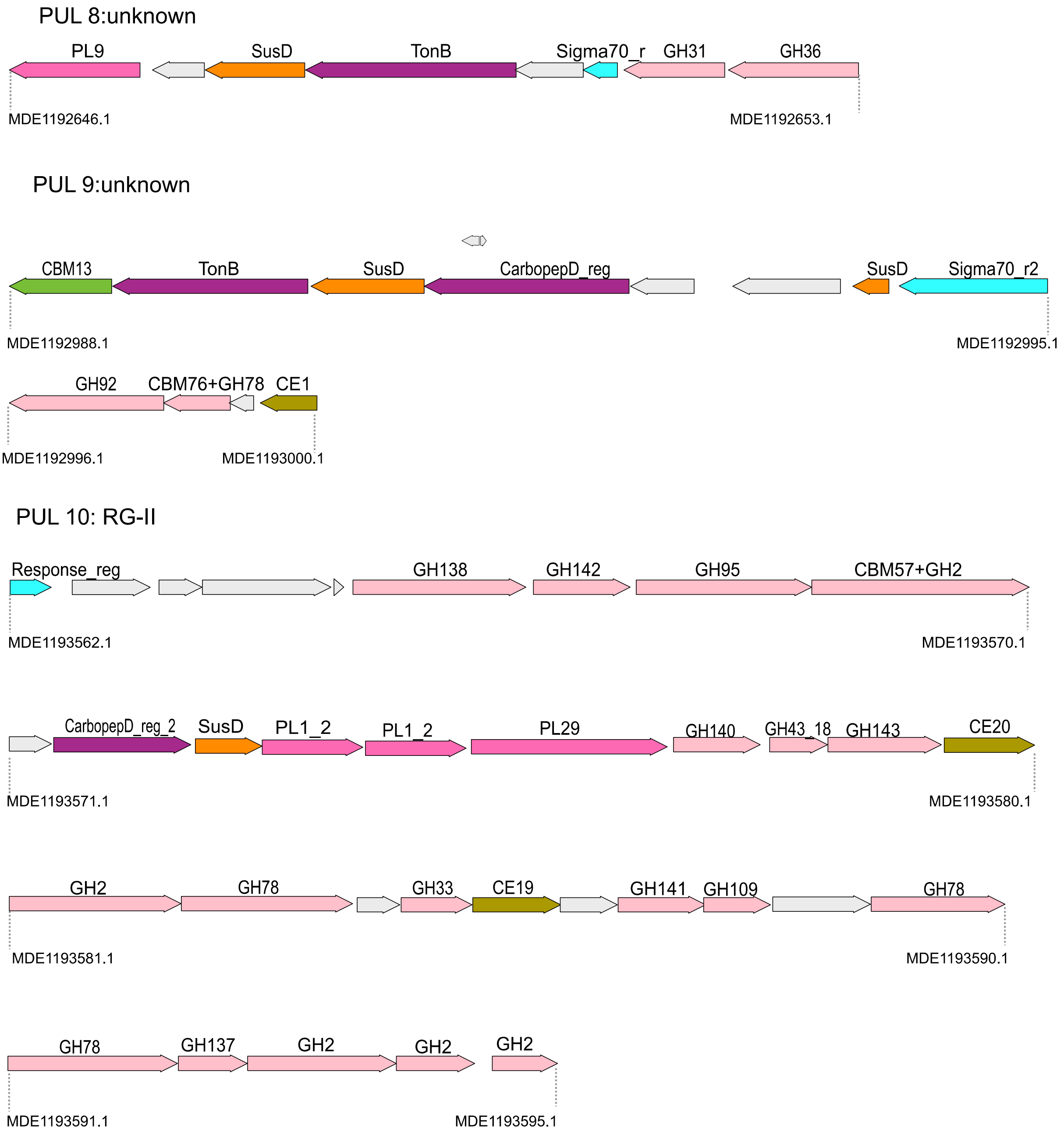

Figure S18c. Putative Polysaccharide Utilization Loci (PULs) identified in MAG13. The PUL-encoded genes are colored according to putative function, and gray arrows indicate both putatively annotated proteins and proteins of unknown function. Representative polysaccharide structures are shown close to each identified PUL.

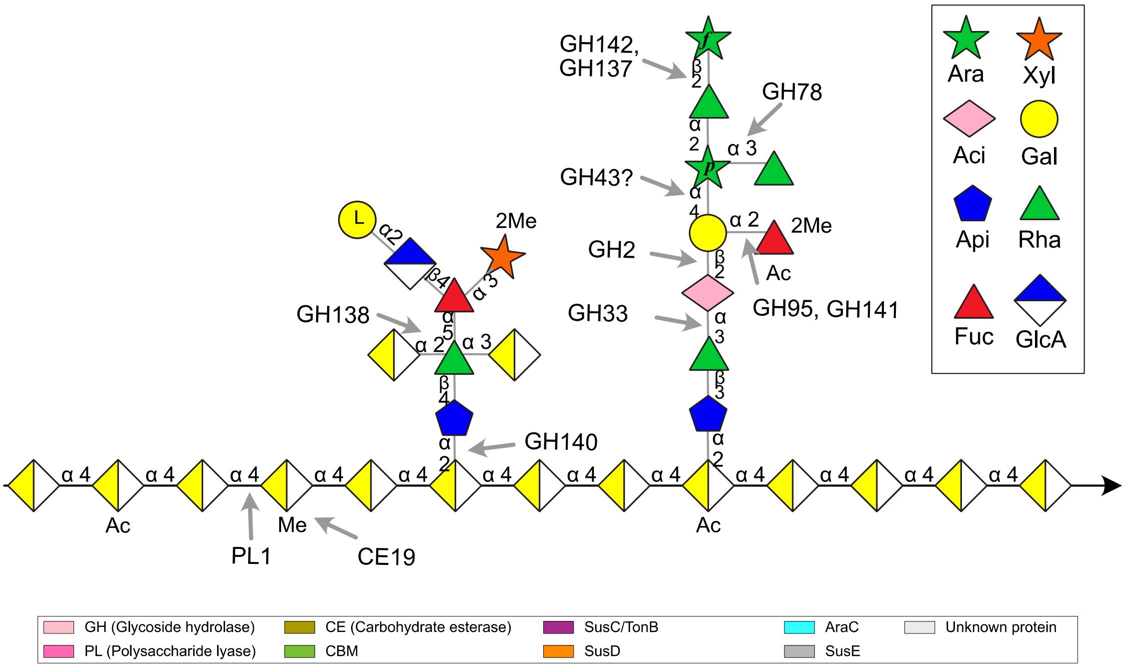

Figure S18d. Putative Polysaccharide Utilization Loci (PULs) identified in MAG13. The PUL-encoded genes are colored according to putative function, and gray arrows indicate both putatively annotated proteins and proteins of unknown function. Representative polysaccharide structures are shown close to each identified PUL.

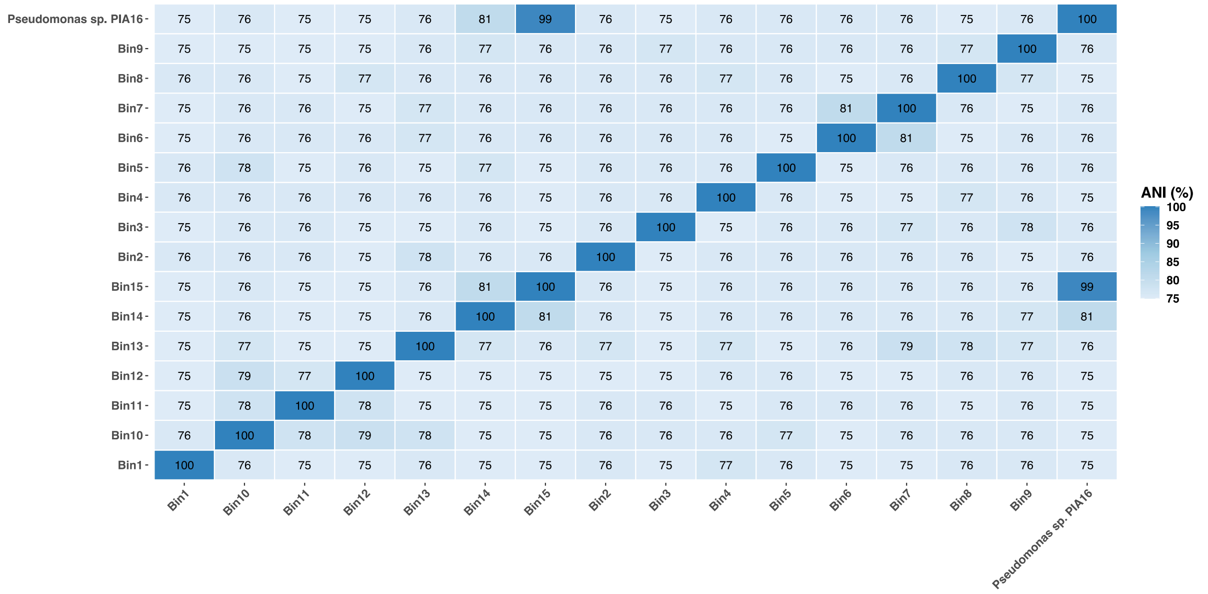

Figure S19. Average nucleotide identity (ANI) between the extracted MAGs (Bins) from the two-week bark metagenome assembly and the isolate *Pseudomonas sp*. PIA16 (*Pseudomonas abieticivorans* sp. nov).

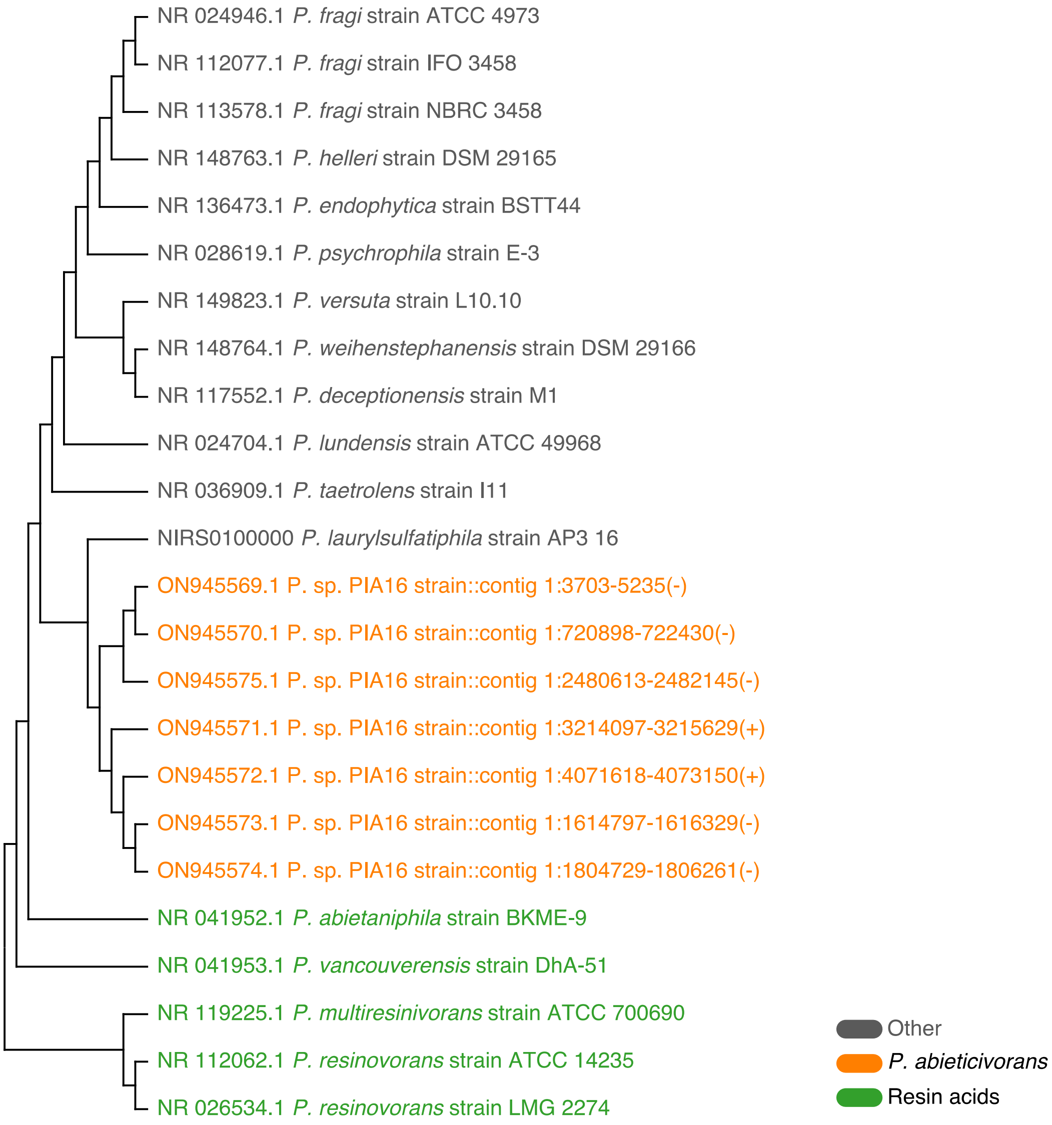

Figure S20. Phylogenetic tree based on the 16S rRNA gene sequences of *P. abieticivorans* (indicated in orange as *P. sp.* PIA 16) and the ten phylogenetically most closely related members of *Pseudomonas* from NCBI: GenBank, in black. Five species known to metabolize resin acids are shown in green.

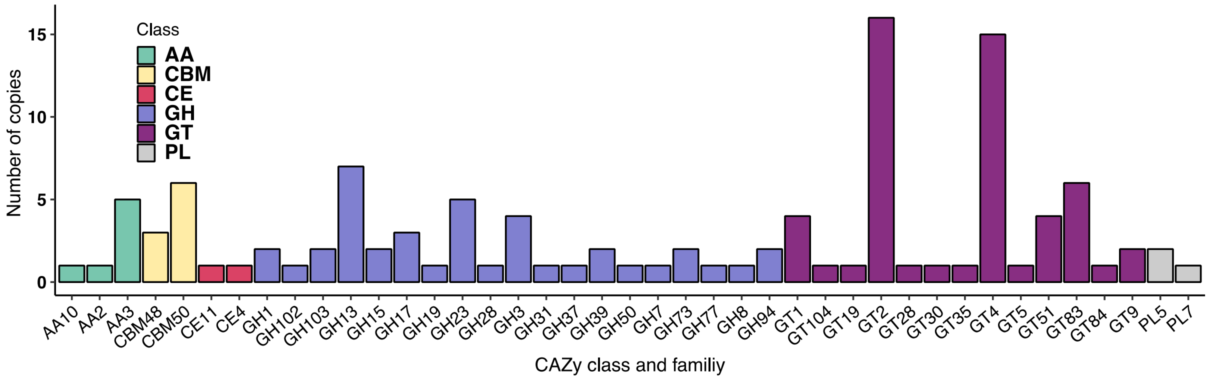

Figure S21. CAZy families and copy numbers putatively encoded by *Pseudomonas abieticivorans*. AA – auxiliary activity, CBM – carbohydrate binding module, CE – carbohydrate esterase, GH – glycoside hydrolase, GT – glycosyltransferase, PL – polysaccharide lyase.

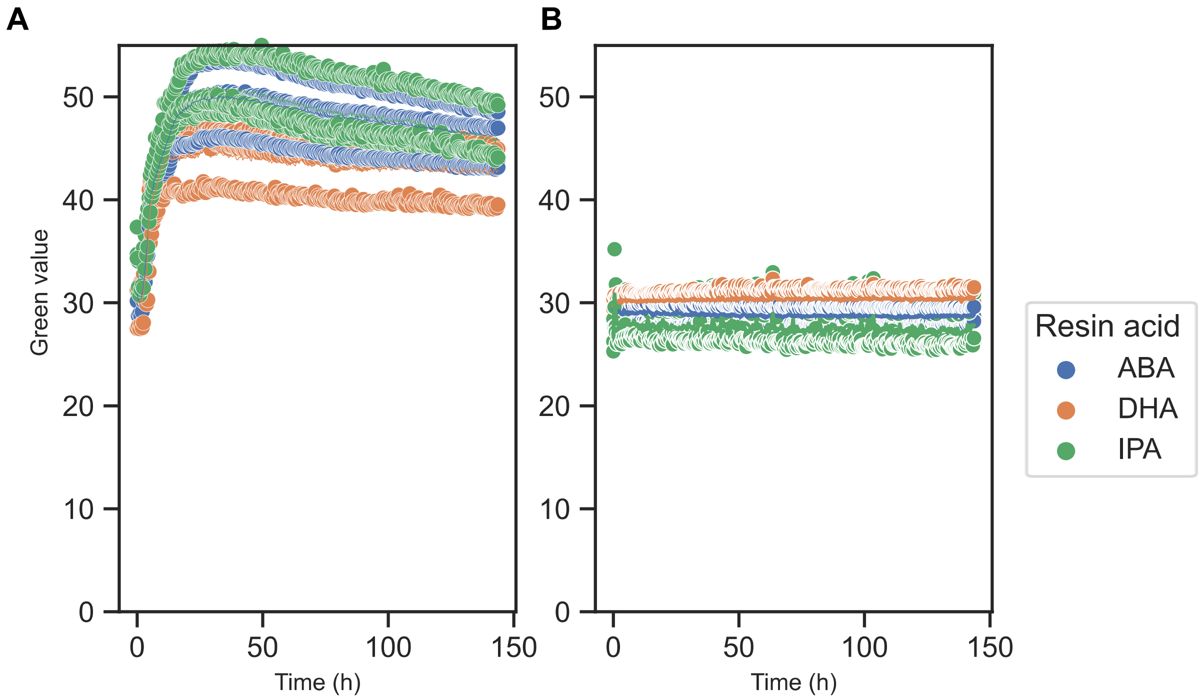

Figure S22. Growth profiles of *P. abieticivorans* on abietic acid (AA), isopimaric acid (IPA), and dehydroabietic acid. Growth Profiler Green Values corresponding to growth based on pixel counts. A) of *P. abieticivorans* B) non-inoculated blank sample. The growth profiles are shown as individual values.

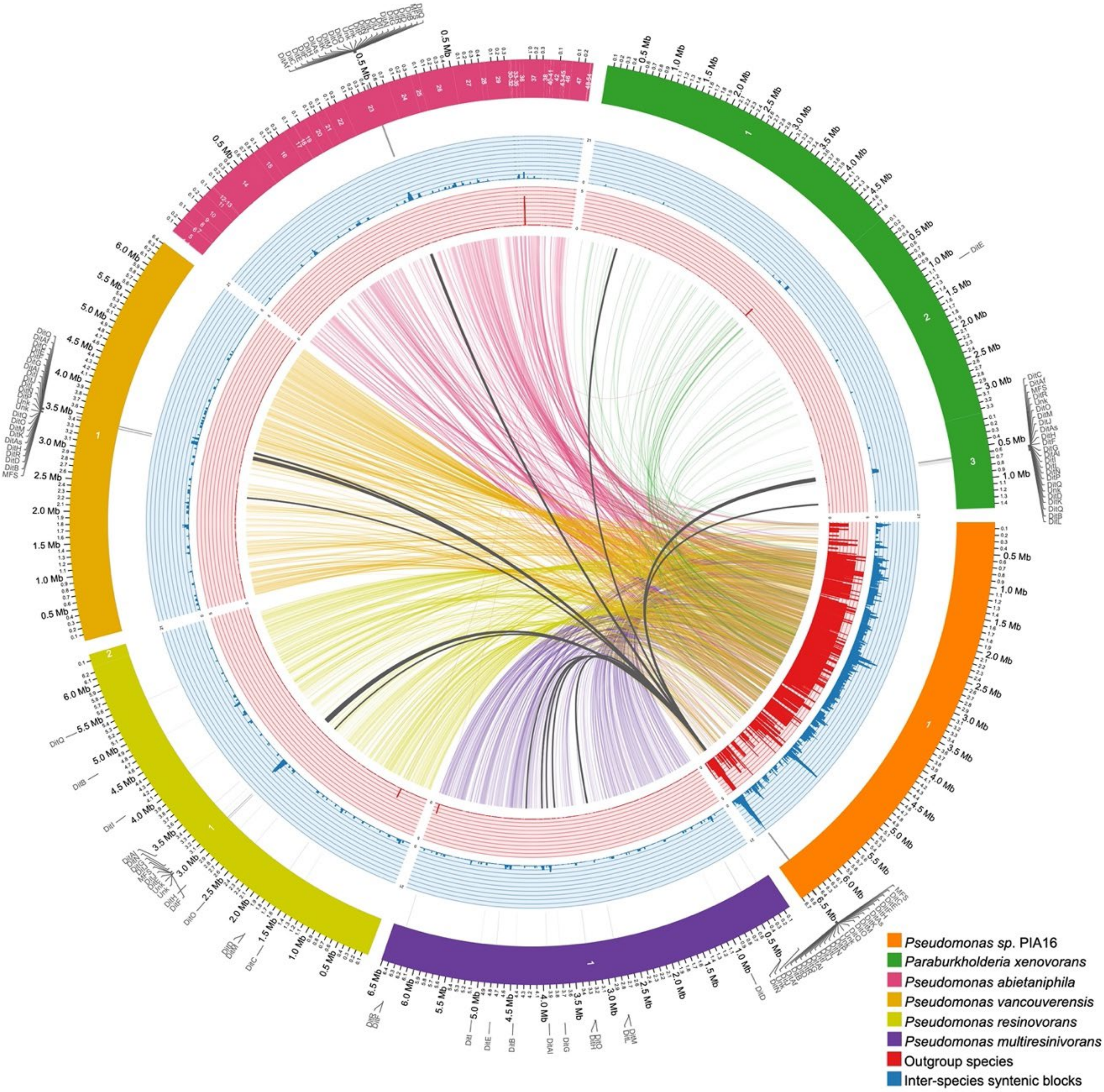

Figure S23. Collinear analysis of five known resin acid utilizing strains and *P. abieticivorans* (sp. PIA16). Numbers inside the ideograms represent chromosome number, plasmid (two in *P. resinovorans*), or contigs (in *P. abietaniphila*). Links between genomes represents coding sequences in synteny blocks. Dark gray links indicate *dit* cluster genes.

Supplementary References

1 Martin, V. J. & Mohn, W. W. Genetic investigation of the catabolic pathway for degradation of abietane diterpenoids by *Pseudomonas abietaniphila* BKME-9. *Journal of bacteriology* **182**, 3784-3793 (2000).

2 Smith, D. J., Martin, V. J. & Mohn, W. W. A cytochrome P450 involved in the metabolism of abietane diterpenoids by *Pseudomonas abietaniphila* BKME-9. *Journal of bacteriology* **186**, 3631-3639 (2004).

3 Smith, D. J., Park, J., Tiedje, J. M. & Mohn, W. W. A large gene cluster in *Burkholderia xenovorans* encoding abietane diterpenoid catabolism. *Journal of bacteriology* **189**, 6195-6204 (2007).

4 Carrión, O., Miñana-Galbis, D., Montes, M. J. & Mercadé, E. *Pseudomonas deceptionensis* sp. nov., a psychrotolerant bacterium from the Antarctic. *International Journal of Systematic and Evolutionary Microbiology* **61**, 2401-2405 (2011).

5 von Neubeck, M. *et al. Pseudomonas helleri* sp. nov. and *Pseudomonas weihenstephanensis* sp. nov., isolated from raw cow's milk. *International Journal of Systematic and Evolutionary Microbiology* **66**, 1163-1173 (2016).

6 Furmanczyk, E. M., Lipinski, L., Dziembowski, A. & Sobczak, A. Genomic and functional characterization of environmental strains of SDS-Degrading *Pseudomona*s spp., providing a source of new sulfatases. *Frontiers in Microbiology* **9**, 1795 (2018).

7 Silverstein, R. M., Webster, F. X., Kiemle, D. J. & Knovel. *Spectrometric Identification of Organic Compounds*. (Wiley, 2005).
