# Supplementary material for: Resin acids play key roles in shaping microbial communities during degradation of spruce bark
Source: Nat Commun. 2023 Dec 9;14:8171. doi: 10.1038/s41467-023-43867-y (PMC10710418; doi:10.1038/s41467-023-43867-y)
Supplement: Supplementary file 3 — Reporting Summary [file 41467_2023_43867_MOESM3_ESM.pdf]

nature portfolio

Corresponding author(s): Johan Larsbrink

Last updated by author(s): Nov 15, 2023

Reporting Summary

Nature Portfolio wishes to improve the reproducibility of the work that we publish. This form provides structure for consistency and transparency in reporting. For further information on Nature Portfolio policies, see our Editorial Policies and the Editorial Policy Checklist.

Statistics

For all statistical analyses, confirm that the following items are present in the figure legend, table legend, main text, or Methods section.

n/a	Confirmed	
☐	☒	The exact sample size (*n*) for each experimental group/condition, given as a discrete number and unit of measurement
☐	☒	A statement on whether measurements were taken from distinct samples or whether the same sample was measured repeatedly
☐	☒	The statistical test(s) used AND whether they are one- or two-sided<br>*Only common tests should be described solely by name; describe more complex techniques in the Methods section.*
☒	☐	A description of all covariates tested
☐	☒	A description of any assumptions or corrections, such as tests of normality and adjustment for multiple comparisons
☐	☒	A full description of the statistical parameters including central tendency (e.g. means) or other basic estimates (e.g. regression coefficient) AND variation (e.g. standard deviation) or associated estimates of uncertainty (e.g. confidence intervals)
☒	☐	For null hypothesis testing, the test statistic (e.g. *F*, *t*, *r*) with confidence intervals, effect sizes, degrees of freedom and *P* value noted<br>*Give P values as exact values whenever suitable.*
☒	☐	For Bayesian analysis, information on the choice of priors and Markov chain Monte Carlo settings
☒	☐	For hierarchical and complex designs, identification of the appropriate level for tests and full reporting of outcomes
☒	☐	Estimates of effect sizes (e.g. Cohen's *d*, Pearson's *r*), indicating how they were calculated

Our web collection on statistics for biologists contains articles on many of the points above.

Software and code

Policy information about availability of computer code

Data collection	MinKNOW Core (5.1.0), MinKNOW Release (21.10.4)
Data analysis	Bandage (0.8.1), Barrnap (0.9), BLASTn, BLASTp, CheckM (1.1.3), Chromeleon (7.2.10), CLUSTALW with msa package, CoverM (0.6.1), Cutadapt (3.7), Cutadapt (2.8), dbCAN2 HMMdb (10), eggNOG-mapper, Filtlong (0.2.0), Flye (2.9), Genome Taxonomy Database toolkit (2.1.0), Genome Taxonomy Database toolkit (1.7.0), Guppy (6.1.5), Kaiju (1.8.2), Krona (2.8.1), Masshunter, MCScanX, R. Circos (0.69-9), Medaka (1.6.1), Medaka (1.4.3), Megahit (1.2.9), Minimap2 (2.24r1122), MinKNOW guppy (g.6.5.7), MIS Standard Software, NanoPlot (1.36.2), NanoStat (1.6.0), NIST MS Search Programme (2.2), Porechop (0.2.4), Prokka (1.14.6), Racon (1.4.20), Racon (1.4.13), Rasusa (0.6.1), samtools (1.14), Usearch11, WebMGA. RStudio IDE (2023.3.0.386) and packages ampvis2 (2.8), tidyverse (2.0.0), seqinr (4.2.30), ShortRead (1.58.0), iNEXT (3.0.0), mmgenome2 (2.1.3), bios2msd, adegenet, ggtree, ggplot2, ape.<br>All the datasets and code used in this study for phylogenetics 16s rRNA and collinearity analysis are available at DOI:10.5281/zenodo.7596797.

For manuscripts utilizing custom algorithms or software that are central to the research but not yet described in published literature, software must be made available to editors and reviewers. We strongly encourage code deposition in a community repository (e.g. GitHub). See the Nature Portfolio guidelines for submitting code & software for further information.

Data

Policy information about availability of data

All manuscripts must include a data availability statement. This statement should provide the following information, where applicable:

- Accession codes, unique identifiers, or web links for publicly available datasets
- A description of any restrictions on data availability
- For clinical datasets or third party data, please ensure that the statement adheres to our policy

All sequencing reads have been deposited at the National Center for Biotechnology Information (NCBI) under BioProject ID PRJNA912085 and PRJNA803013. The 16S and ITS reads have been deposited in the Sequence Read Archive (SRA) under the accession numbers SRX21853827-SRX21853858 (Table S12). PIA16 reads have been deposited under the accession numbers SRR24684300 and SRR24684301 (Biosample accession SAMN25609858), and the metagenome reads under accession numbers SRR22729505 and SRR22729506 (Biosample accession SAMN32218794). The recovered MAGs have been deposited in GenBank under the accession numbers SAMN32241433-SAMN32241447 (Table S13). The P. abieticivorans PIA16 genome has been deposited in GenBank under the accession number GCA_023509015.1, the 16S rRNA sequence under the accession number ON945571.1, and the rpoD sequence under the accession number OP594298. Other data generated or analyzed during this study are included in the Supplementary Information files, and Source data are provided with this paper.

Research involving human participants, their data, or biological material

Policy information about studies with human participants or human data. See also policy information about sex, gender (identity/presentation), and sexual orientation and race, ethnicity and racism.

Reporting on sex and gender	N/A
Reporting on race, ethnicity, or other socially relevant groupings	N/A
Population characteristics	N/A
Recruitment	N/A
Ethics oversight	N/A

Note that full information on the approval of the study protocol must also be provided in the manuscript.

Field-specific reporting

Please select the one below that is the best fit for your research. If you are not sure, read the appropriate sections before making your selection.

☒ Life sciences ☐ Behavioural & social sciences ☐ Ecological, evolutionary & environmental sciences

For a reference copy of the document with all sections, see nature.com/documents/nr-reporting-summary-flat.pdf

Life sciences study design

All studies must disclose on these points even when the disclosure is negative.

Sample size	The spruce bark sample was sourced from the forestry company Holmen AB, including several kilos of spruce bark. The sample was milled and mixed to create a more homogeneous sample that then was composed of bark from multiple trees, estimated to be around 70 years of age at harvest.
Data exclusions	No data were excluded in the study.
Replication	The growth experiments were conducted over six months with duplicate samples and corresponding analyses for each timepoint as well as corresponding control (abiotic) samples. Technical replicates were used also in the analyses of bark components and sequencing. Replicate analyses showed good agreement among the samples.
Randomization	Randomization was not used or needed for the experimental setup as it did not involve study groups, but rather one large and long-term experiment.
Blinding	No blinding was needed for these kinds of experiments that do not involve study groups.

Reporting for specific materials, systems and methods

We require information from authors about some types of materials, experimental systems and methods used in many studies. Here, indicate whether each material, system or method listed is relevant to your study. If you are not sure if a list item applies to your research, read the appropriate section before selecting a response.

Materials & experimental systems

n/a	Involved in the study
☒	☐ Antibodies
☒	☐ Eukaryotic cell lines
☒	☐ Palaeontology and archaeology
☐	☒ Animals and other organisms
☒	☐ Clinical data
☒	☐ Dual use research of concern
☒	☐ Plants

Methods

n/a	Involved in the study
☒	☐ ChIP-seq
☒	☐ Flow cytometry
☒	☐ MRI-based neuroimaging

Animals and other research organisms

Policy information about studies involving animals; ARRIVE guidelines recommended for reporting animal research, and Sex and Gender in Research

Laboratory animals	No laboratory animals were used in the study.
Wild animals	No animals were captured or studied, only microorganisms.
Reporting on sex	N/A, only microorganisms studied.
Field-collected samples	Samples were not collected in the field, but came from a forestry company.
Ethics oversight	NA, only microorganisms studied.

Note that full information on the approval of the study protocol must also be provided in the manuscript.

Plants

Seed stocks	N/A
Novel plant genotypes	N/A
Authentication	N/A
